# “We struggle with the earth everyday”: parents’ perspectives on the capabilities for healthy child growth in *haor* region of Bangladesh

**DOI:** 10.1186/s12889-020-8196-9

**Published:** 2020-01-31

**Authors:** Barnali Chakraborty, Sepideh Yousefzadeh, Shrinivas Darak, Hinke Haisma

**Affiliations:** 1 BRAC, 75, Mohakhali, Dhaka, Bangladesh; 2 BRAC James P Grant School of Public Health, BRAC University, 5th Floor, (Level-6), icddr,b Building, 68, Shahid Tajuddin Ahmed Sharani, Mohakhali, Dhaka, 1212 Bangladesh; 3 Population Research Centre, Faculty of Spatial Sciences, University of Groningen, Landleven 1, 9747AD Groningen, the Netherlands; 4 Campus Fryslan, University of Groningen, Groningen, the Netherlands; 5 Prayas Health Group, Pune, Maharashtra 411 004 India

**Keywords:** *Haor*, Child growth, Capability approach

## Abstract

**Background:**

Childhood stunting is an important public health problem in the *haor* region of Bangladesh. *Haor* areas are located in the north-eastern part of the country and are vulnerable to seasonal flooding. The key objective of this study is to identify the capabilities of the parents and their children that shape multidimensional child growth outcomes in the *haor* region in the first thousand days of life.

**Methods:**

A qualitative study was conducted in two sub-districts of the *haor* region, including in *Derai* in the *Sunamganj* district and *Baniachang* in the *Habiganj* district. We facilitated eight focus group discussions with the parents of children under age two. To allow us to explore individual stories, we conducted in-depth interviews with four fathers and four mothers. A capability framework to child growth was used in shaping the interview guides and analysing the data.

**Results:**

The findings were categorised at four levels: a) capabilities for the child, b) capabilities for the mother, c) capabilities for the father, and d) capabilities at the household level. At the child’s level, the parents discussed the capability to stay away from disease and to eat well, the capability to stay happy and playful, and the capability to be born with God’s blessings and the hereditary traits needed to grow in size. The mothers frequently mentioned the capability to stay healthy and nourished, to stay away from violence, and to practice autonomy in allocating time for child care. The fathers stressed the earning opportunities that are affected by long-term flooding and the loss of agricultural productivity. At the household level, they discussed the capability to live in a safe shelter, to be mobile, to overcome their struggles with the earth, and to have a source of safe drinking water.

**Conclusions:**

The capability framework for child growth helped identify relevant capabilities in the *haor* region. These findings can guide discussions with communities and policy makers about developing programmes and interventions aimed at enhancing the identified capabilities for child growth in this vulnerable region.

## Background

**Fig. 1:**
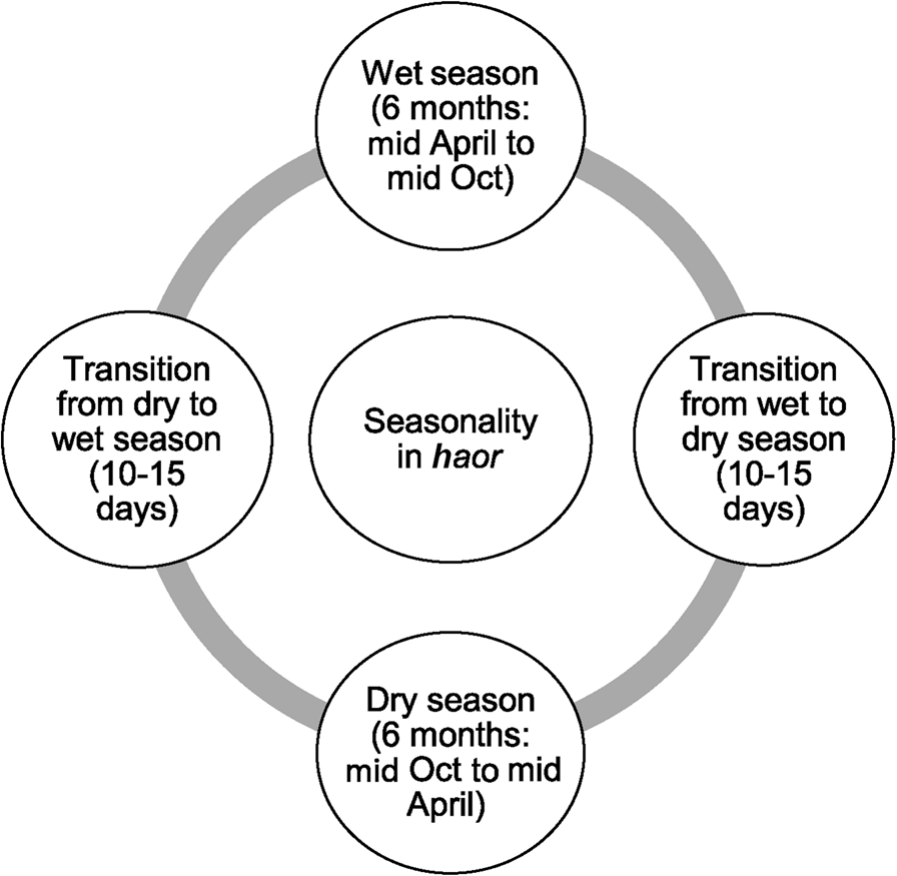
Seasonal cycle of haor areas

Stunting is a crucial indicator in efforts to alleviate childhood undernutrition in developing countries. If the threat of stunting is not addressed at the earliest possible point in the life course, preferably during the first thousand days of life (from conception until 2 years of age), its effects on an individual’s physical and cognitive development may be irreversible [1, 2]. Growth retardation at the foetal stage (in the form of SGA: small for gestational age) accounts for 12% of child deaths and contributes to chronic stunting if the new-born survives [1]. The problem of childhood stunting is rampant across Bangladesh, and particularly in the *haor* region of the country. About 46% of the children under age five who live in the *haor* experience stunting [3]. The *haor* is a wetland ecosystem located in the north-eastern part of the country, surrounded by the hill ranges of Meghalaya (India) to the north, the hills of Tripura and Mizoram (India) to the south, and the highlands of Manipur (India) to the east. These areas are physically vulnerable because of frequent seasonal flooding. There are two main seasons in the region: wet and dry, which are accompanied by two transition phases from the wet to dry and the dry to wet seasons (Fig. 1). During the wet season, the land mass is submerged by run-off rain water that flows from upstream, and remains under water for half of the year.

The surveys and studies conducted in the *haor* region reveal that more than half of the population are poor, marginal farmers, and that the people in the region score relatively low on a range of well-being markers [3–5]. For example, whereas the proportions for markers of adult literacy, undernourished pregnant women (MUAC: Mid Upper Arm Circumference), undernourished non-pregnant adult women (BMI: Body Mass Index) appropriate infant and young child feeding practices, and access to improved latrine are, respectively, 56, 18, 17, 23%, and 40–45% at the national level; the corresponding proportions in the *haor* are 37, 33, 24, 11%, and 27–39% [3, 5–8]. The aforementioned markers have been recognized as important explanatory variables for the persistent problem of childhood stunting in Bangladesh. In particular, the importance of maternal BMI and short stature in relation to childhood stunting has been well documented in the context of Bangladesh [9–11]. Increasingly, there is an added focus on other biological, socio-cultural, and environmental determinants of stunting [12–16]. However, the complexity of child growth in context of haor is still only partly understood. In this paper, we attempt to contribute to this information gap.

**Fig. 2:**
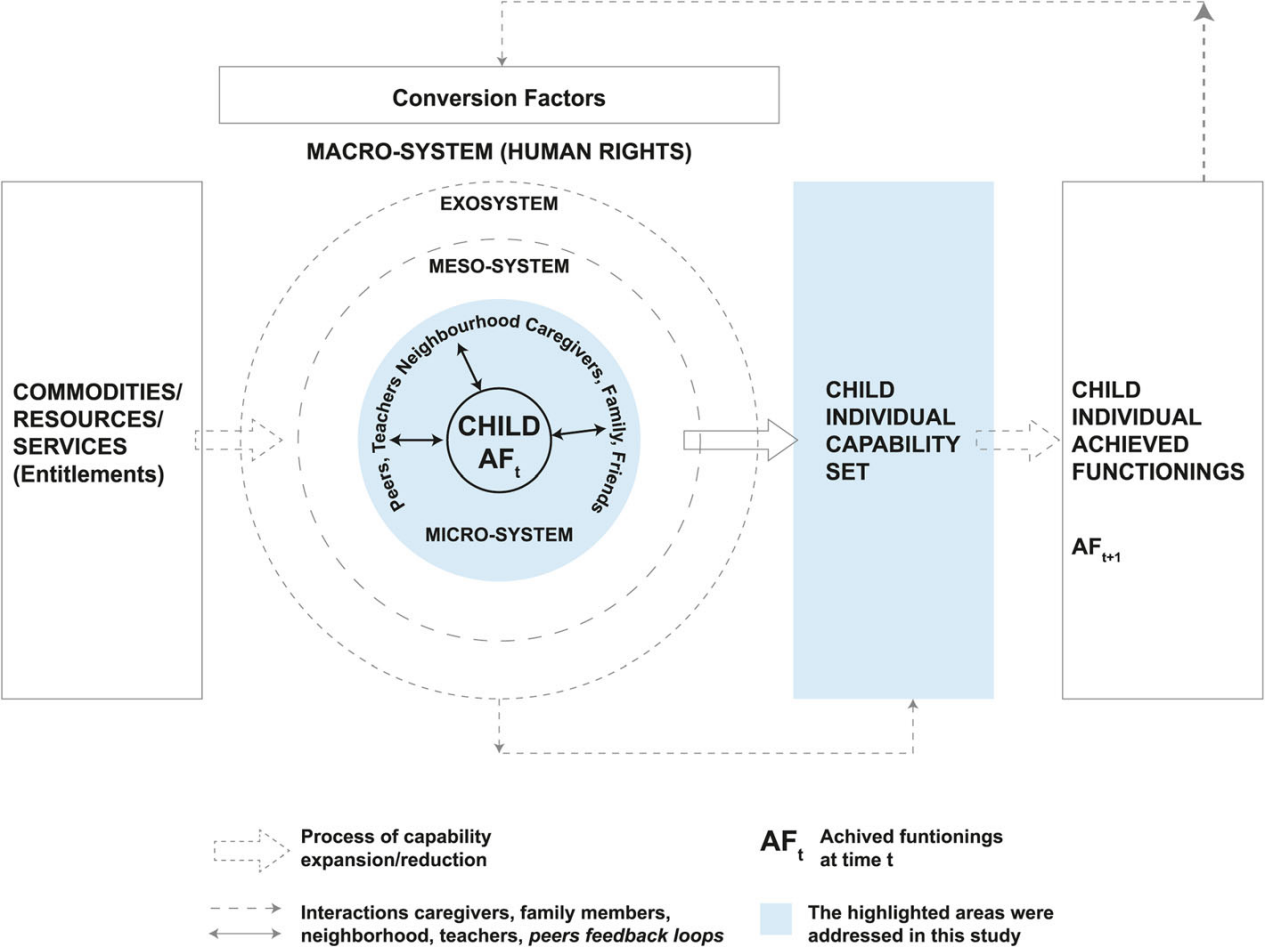
A capability approach to child growth (adapted from Yousefzadeh et al. 2018 [21])

Multiple approaches for explaining poor child growth have been proposed and applied by experts and scholars [17–20]. The most widely used of these approaches is UNICEF’s conceptual framework in which growth is defined as an anthropometric outcome led by multifactorial determinants. The determinants are described at the immediate level (dietary intake and health status), the underlying level (status of food security, feeding and caring, availability of services, education) and the basic level (agricultural, political and economic context) [20]. Haisma et al. 2017 [17], by contrast, proposed using Amartya Sen’s capability approach (CA) as a multi-dimensional framework for child growth [17]. In this framework, child growth is defined as the achievement of a certain set of capabilities of the children and their parents that include the typical dimensions related to nutrition and physical growth. However, other dimensions, such as shelter and love and care, are also included in the aggregated outcome. Thus, this framework differs from the above mentioned UNICEF framework(s) in several ways: its outcome is multi-dimensional; it captures contextual differences; and it concentrates on people’s opportunities rather than the outcomes per se [17]. Building on Haisma et al.’s definition of child growth, the capability framework to child growth (CFCG) was developed by Yousefzadeh et al. in (2018) [21] order to conceptualise the development of children’s capabilities or opportunities within their periphery for achieving wellbeing outcomes [21]. In this study, we use the CFCG framework (Fig. 2) to examine the capabilities of child growth in the *haor* region, while focusing on the first thousand days of life.

According to the CFCG, capabilities represent the various sets of options or opportunities from which an individual chooses in order to do or achieve something that s/he values [21, 22]. The periphery in which the children grow up encompasses multiple layers of relationships across micro, meso, exo, and macro systems [21]. The micro system represents the proximate layer at which caregivers and family members play important roles in shaping children’s capabilities to grow well. The meso system (the relationships between caregivers, doctors, or teachers), the exo system (the relationships across two or more settings that influence the child’s development indirectly), and the macro system (belief systems, social norms, culture, and institutional settings) are connected through subsequent layers [21]. Children draw their entitlements from the micro systems through their proximate relationships with their parents or caregivers [17, 21, 23]. Therefore, children’s capabilities are dependent on their parents’ capabilities or external capabilities, which they receive through intergenerational transfers [21, 24, 25]. The second element of this framework includes the achieved outcomes of the children that are primarily influenced by the choices of the parents. The children are, however, capable of influencing their parents’ agency through their body language; such as by crying or smiling [21]. Thus each child’s developmental process is influenced by the interaction of individual and external capabilities, the agencies of the child and her/his parents, and the child’s achieved outcomes. The other two elements of this framework are resources and conversion factors. Resources are the means for achieving something (e.g., intelligence, income, or the availability of essential welfare services); whereas conversion factors are facilitators of or barriers to converting the resources into individual capabilities and outcomes. Conversion factors can be internal (e.g., physical conditions, sex, knowledge, skills), societal (e.g., traditions, social norms, power relations), and environmental (e.g., climate, geographical infrastructure) [26, 27].

We apply the CFCG while restricting our focus to the capabilities of the children and their parents that shape child growth outcomes in areas of the *haor* region. The key objective of this study is to identify the child and the parental capabilities that shape the growth outcomes of children under age two.

## Methods

### Study area and population

Two sub-districts, Derai in the *Sunamganj* district and *Baniachang* in the *Habiganj* district, were selected purposively for the study. Both areas are dominated by *haors* of varying depths*.* Deep *haors* are subject to heavy flooding, and the waves that form in the waters that collect in these *haors* can be very high during monsoons, when the wind is strong. The population of Derai is around 244 thousand, and the population of Baniachang is more than 300 thousand [28]. Most of the people living in these areas attempt to safeguard their houses from flooding by building them slightly above the ground in clusters known as ‘*hati*’ (field notes). Each *hati* contains 20 to 50 houses. BRAC, a global NGO, has a stable field setup in these areas that seeks to help the vulnerable communities through integrated development interventions. These interventions include providing microfinance, a boat school programme, health and nutrition counselling, and sanitation and hygiene facilities (e.g., tube wells and toilets). In our study, we relied on the BRAC platform to gain entry to the local communities.

### Study design and recruitment of the participants

A qualitative study was conducted with both parents (fathers and mothers) of children under age two. The study was designed using the qualitative research cycle described by Hennink et al. 2010 [29]. In the design cycle, we used the CFCG framework for the development of the interview and focus group guides [21]. Operationalization of this framework, applying a multidimensional lens shaped the interview guides in a broader way through expanding the focus from biological to societal and contextual dimensions. In the data collection cycle, we made sure that deeper insights into emic perspectives of participants were gained through an iterative process of interviewing and reflection. For each of the following interviews or focus groups we reflected on the previous ones, and would identify where we want to go deeper in the next interview or focus group using probing techniques for the questions included in the interview guides. In the analytic cycle, we used both inductive and deductive coding. Initially, we applied grounded theory to identify the emic perspectives of the respondents [30]. The inductive codes would then be deductively interpreted against the concepts of the capability approach to identify relevant capabilities.

Eight focus group discussions (FGD) were facilitated: four with the mothers and four with the fathers. For each FGD, six to eight participants were recruited. To explore individual stories in greater detail, in-depth interviews (IDI) were conducted with four fathers and four mothers. The main purpose of conducting in-depth interviews was to give participants adequate privacy when sharing stories on topics like domestic violence or individual agency. The numbers of IDI and FGD were determined based on the level of saturation achieved at different stages of data collection. In the first round, two focus group discussions and four IDIs were conducted with the mothers and the fathers. These discussions and interviews were immediately transcribed and checked for variation in the data in alignment with the research objectives [29]. In the second round, more data were collected. This process of data collection continued until the responses of the participants to the research questions no longer contained any new stories; i.e., until data saturation was achieved.

Fathers and mothers from households with children under age two who expressed willingness to take the time to participate in an interview or a focus group discussion were considered eligible to participate in the study. To ensure diversity in the information collected, the fathers and the mothers selected were from different households. The local field assistants of the BRAC field office helped the research team find the locations and the households from which the participants were selected. The IDI and the FGD were conducted in community spaces or homes that provided sufficient privacy.

### Data collection, coding and analysis

An interview guide with open-ended questions shaped the FGDs and the IDIs (see Additional file 1). The process of preparing and shaping the interview guides included conducting a review of the existing literature on capability approach, children’s growth and wellbeing, engaging in a series of consultations with academic experts on capabilities and child growth, and pilot testing in the study areas [20, 21, 23, 24]. To identify the capabilities, the participants were asked what kinds of capabilities or qualities a mother or a father needs to have to take good care of her/his children or to keep them healthy and well-nourished; and why they consider those capabilities important. The participants were also asked how they determine whether their child is or is not growing well. The principal researcher conducted one-to-one IDIs and FGDs facilitated by a note taker. The FGDs focused on collecting information about the opinions held by the community rather than about personal experiences.

The interview notes were tape recorded and transcribed verbatim in order to capture the meaning of all of the participants’ verbal and non-verbal expressions. The domains explored through FGDs (more of opinions) and in-depth interviews (more person narratives and experiences) were similar which led to similar set of deductive codes, coming mainly from our framework of capabilities approach. Therefore, a single comprehensive codebook was prepared which was used to code the transcripts of FGD as well as IDIs (Additional file 1). The codebook was developed by coding first few transcripts of IDI and FGD. This code book was then reviewed to check for duplication of codes and the operational definition of these codes by the principal researcher which was then shared and discussed with other co-authors. Tabulation of deductive and inductive codes was done to check for the new information emerging from the data. Coding was done using N-Vivo Software (QSR International Pty Ltd., Australia).

In identifying the capabilities, we considered all the responses appeared relevant to capabilities, the participants indicated they valued or cited to justify their actions in achieving multidimensional child growth outcomes. Codes served as the basic unit of fragmenting data. Code families including ‘parent node’ (broader code) and ‘child node’ (sub-code) were created to segment or categorize specific issues under the broader code. Quotations pertaining to relevant codes and code families were retrieved and read carefully to understand the underlining concepts. Categorization and comparison were mainly used to interpret the data. The coded data were retrieved using N-Vivo Software to check, review and analyze the responses or quotes whenever needed. Scientific rigour was obtained through the iterative process of inductive and deductive reasoning. The application of grounded theory where codes are developed inductively, staying close to the actual textual data, followed by a deductive coding round applying the concepts of the capability approach ensures that findings are grounded in the data [29, 30]. The interpretation is supported by the quotes presented.

## Results

### Background characteristics of the participants

Most of the participants identified their religious affiliation as Muslim; a few were Hindu. Most of the fathers reported that they work in agriculture (rice cultivation) during the growing (dry) season, while most of the mothers said they perform household work. The fathers indicated that they are jobless or migrate to other towns to engage in small business activities (e.g., cosmetics, masonry) during the wet season. Most of the participants had incomplete primary education, and their literacy level was confined to being able to sign their own name. A brief description of the background characteristics of the participants is provided in Table 1.

**Table 1:** Background characteristics of the participants

Mothers	IDI	FGD	Fathers	IDI		FGD	
Age range	23–30 years	16–35 years	Age range	21–45 years			
Religion							
Muslims	4	26	Muslims	3		23	
Hindus		7	Hindus	1			
Occupation				Dry season	Wet season	Dry season	Wet season
Household work	3	32	Rice Cultivation	2		18	
Others	1 (Stitching)	1 (grocery)	Small Business (selling vegetables, betel leaves and nuts, and cosmetics; fishing/fish selling)	2	4	5	9
			Day labourer (masonry, pulling *rickshaws,* rowing boats)				8
			jobless				6
Education							
No education		1	No education	1		5	
Only Signature	3	15	Only Signature	1		8	
Below Primary	1	3	Below Primary	1		5	
Primary education		9	Primary education	1		2	
Below SSC (secondary school certificate)		3	Below SSC (secondary school certificate)			3	
SSC		2					

### Capabilities for healthy child growth

The capabilities that emerged from the data are summarised in Table 2. Many of these capabilities are interrelated, and the participants expressed them as a single capability or as interrelated capabilities. To maintain this interrelation, some of these capabilities may appear in several places or in combination.

**Table 2:** List of capabilities for achieving healthy child growth outcomes

Children	Mothers	Fathers	Household
*Being able to stay away from disease and eat well*	*Being able to stay healthy and eat well*	*Being able to earn in all seasons and provide the family with the things they need*	*Being able to live in a safe home during the wet season*
*Being able to be born with God’s blessings and the hereditary traits needed to grow in size*	*Being able to stay away from domestic violence*	*Being able to save the future and the one who creates the future*	*Being able to overcome struggles with the earth to stay neat and clean*
*Being able to stay happy and playful*	*Being able to allocate time for child care as desired*	*Being able to express love by purchasing toys for the children and other items as required*	*Being able to be mobile in different seasons*
	*Being able to be engaged in income- generating activities*	*Being able to be educated in order to get a job and educate the children*	*Being able to secure a source of safe drinking water for the family*
	*Being able to express love (maya-mohabbat) and take care of the children*	*Being able to be healthy, energetic, have a brain (intelligence) and be religious*	
	*Being able to be educated in order to provide the children with good care*		

### Child capabilities

#### Being able to stay away from disease and eat well

According to the participants, a child who is in good health and is free of disease eats well and, as a result, grows well. When a child smiles, plays, and eats normally s/he is considered healthy; but when a child does not eat, feels sick, frequently suffers from diseases, or cries throughout the day, s/he is considered unhealthy. A mother explained this distinction in the following way:

*“When the baby has any disease it doesn’t grow – for example, my baby is now eating well. When it gets sick it doesn’t eat well, it stops eating.” FGD, Mothers, Sarail, Baniachang.*

It is worth mentioning that whenever the participants talked about health or nutrition they referred to them simultaneously, and explained them as interrelated capabilities. It was, for example, frequently stated that children will grow well if they are fed good foods containing vitamins such as *dal* (pulses), fish, vegetables, fruits as well as rice. A mother summed up this perception in the following way:

*“If we could feed fruits to the children they would have grown faster; but we cannot get fruits for them. Whatever we feed them includes breast milk and some rice.” IDI, Mother, Agua, Baniachang.*

#### Being able to be born with God’s blessings and having the hereditary traits (bongsher dhara) needed to grow in size

A number of the participants said they can tell whether a baby is growing well by looking at the sizes of children of the same or different ages, and especially at the sizes of their faces, fingers, hands, and legs. Some of the participants stressed that whether a baby grows in size and becomes tall or short is determined by God’s will. One of the fathers expressed this perspective as follows:

*“It is Allah’s will; Allah knows when he will make someone tall and whom. We don’t know …*” *FGD, Fathers, Golbogi, Baniachang.*

Some of the participants said they believe that a child’s hereditary background *(bongsher dhara)* determines whether s/he will grow tall or remain short. During a FGD, one of the mothers shared her views on the role of heredity in determining stature:

“*See, everyone has his or her family heredities (bongsher dhara). Within a family there can be tall and short people. For example, my son is short. … … … .. For example, if the father is short and the mother is short then the children will be short. This is hereditary.” FGD, Mothers, Sarail, Baniachang.*

#### Being able to stay happy and playful

Several of the participants stated that looking happy and playing with others indicate that a child is in good health. They said they believe that parents should monitor their children to check whether they are peaceful and joyful, and whether they are playing with others or staying isolated. A father from *Baniachang* talked about the signs parents look for in assessing the health of their children:

“… *we need to look at whether they are experiencing any problems; for example, whether they look peaceful and joyful … and are playing with others as my baby is doing … whether they are staying isolated, whether there are any health problems that these things reveal.” FGD, Fathers, Golbogi, Baniachang.*

### Maternal capabilities

#### Capability to stay healthy and eat well

The participants stated that a mother needs to eat well, stay neat and clean, and stay healthy in order to take good care of her baby. According to them, a mother who stays healthy is likely to have the strength she needs to breastfeed her baby, prepare other foods for the baby, shower and clean the baby, and take care of other family members. One of the fathers explained his views on the importance of maternal health as follows:

*“If the mother is healthy the child will get good care and love. If the baby is a breastfeeding baby, s/he will get adequate* breast milk*. If the mother is able to eat well she will stay healthy and will be able to work hard. She will be able to take care of others. If her health is not good, she will not be comfortable taking care of others. If she feels well then she will be able to take care of the children. That’s why it is important for a mother to be healthy.” IDI,* Father*, Talibpur*, *Baniachang.*

The participants indicated that they see maternal health and nutrition as complementary dimensions for achieving the functioning of child care. They stated that the consumption of vitamins or fruits during pregnancy could make the foetus bigger and lessen the chances of a normal delivery. According to these participants, a rice-based diet is an ideal diet for a pregnant mother, as it will help her to gain the strength she needs to deliver the baby. However, they stressed that following such a diet is not advisable after the delivery as it could interrupt the production of breast milk for the baby.

*“If the mother doesn’t eat, her children won’t be able to get nutrition. If she doesn’t eat how will her breast milk be produced?” FGD, Fathers, Golbogi, Baniachang.*

#### Being able to stay away from domestic violence

Both the fathers and the mothers said they believe that fighting between couples is a normal part of domestic life. However, the women participants in both areas reported that acts of physical or mental abuse committed by a husband or by other family members, such as in-laws, induce fear, and that the physical and mental shock of experiencing such abuse can be unbearable. One of the mothers said in a FGD that her husband broke her hand and tried to kill her by hanging. She added that when she went to the social committee of their village to ask for justice, she was advised to get along with her husband for the sake of her children. She expressed her views on the violence she experienced as follows:

*“No one should be abused like me; it should be stopped in every house. No one should be abused like I was. There is no justice for abused victims in our village, no one will take initiative to solve the problem or provide justice.” FGD, Mothers, Basakarach, Derai.*

The women participants said they value living in a violence-free environment for the sake of their own wellbeing and that of their children. It was pointed out in the discussions that mothers who are exposed to domestic violence may refuse to eat their meals and to take care of their children for a period of time. According to the participants, children who grow up in a conflict-ridden environment are likely suffer from poor mental health because they feel scared, worry about their mother, become detached from their father, and learn the bad manners.

*“It causes harm to the children: they are shocked, become full of fear, and are scared of their father. Then they don’t eat with their father because they are discontented.” FGD, Mothers, Jarulia, Derai.*

#### Being able to allocate time for child care according to their own wishes

Several of the mothers expressed a desire to stay neat and clean and to take good care of their children. They noted, however, that their mother-in-law often makes decisions for them, as they are not financially empowered and depend on their husband for financial support. They said that if they had their own income they would be able to raise their voice and allocate time for child care according to their own wishes. One of the mothers explained her efforts to balance her responsibilities as follows:

*“We always want to stay neat and clean, to take good care of our children. Then our in-laws say if you spend your time taking care of the children who will do the household work? Then we don’t bother about child care so we have time for the household work. See, if we had our own earnings we could argue. Now we need to depend on them, we eat what they provide. If they shout we can’t speak, because those who have money have more value.” FGD, Mothers, Sarail, Baniachang.*

The women reported that they are expected to always listen to their in-laws or their husband. According to these participants, because their husband works hard in the field and they are expected to take full responsibility for the household work, they are careful to have food ready when their husband gets home. The women observed that the burden of household chores leaves them with limited time to care for their children. One of the mothers in a FGD explained this dilemma in the following way:

*“She (mother) doesn’t need to do any household work or anything so that she can take good care of her baby. If she gets busy with lots of work how can she take good care of the child? If they are going to town, don’t we need to cook for and serve them? What will we do? Child care or cooking?” FGD, Mothers, Jarulia, Derai.*

#### Being able to be engaged in income-generating activities

The participants stated that women in the *haor* have limited opportunities to earn money because of a lack of women-friendly job opportunities. They noted that in many areas women work in the garment industry, but that this is not the case in the *haor*. Some of the mothers said that if there were suitable job opportunities for women they would earn some money in order to provide financially for their families, develop themselves, raise their voices, and achieve peace for their children. The women in one of the FGD discussed these issues in the following way:

*“See we the women here practice pardah (wearing a veil). If there were a garment factory we could work there, … We want to develop ourselves gradually. We want to develop ourselves so that we can move ahead and achieve peace for our children.” FGD, Mothers, Sarail, Baniachang.*

#### Being able to express love (*maya-mohabbat*) and to take care of their children

The participants mentioned that mothers in the *haor* are expected to make every effort to keep their babies well, healthy, and comfortable. Some of the fathers mentioned that a mother in the *haor* should be able to provide more than 60% of the care a baby needs (locally expressed on the scale of 16 *Anna*; i.e., 10 *Anna* out of 16 *Anna*). They said they believe that a mother must have *maya-mohabbat* (maternal affection), which will inspire her to express love for the baby, carry the baby, clean and change his/her nappy, shower and massage him/her with oil or lotion, and feed the baby as required. A mother from Baniachang described the maternal care she provides in the following way:

*“I am here, the mother of the child! The mother needs to feed the baby, shower the baby, help the baby urinate, carry the baby, and so on. All of the tasks involved in keeping the baby well, keeping the baby healthy and comfortable, all are done by me. Will anyone do for my baby what I do? Will my husband do it?” IDI, Mother, Mohammadpur, Baniachang.*

#### Being able to get an education and provide good child care

The participants described getting institutional education as an important capability for a mother that enables her to care for her children efficiently. They said they believe that if a mother is educated, she will be more knowledgeable about feeding, cleaning, and educating her children. Some of the participants also said that having an education is less important for a father than for a mother because the mother is the principal caregiver.

*“An educated father is not essential, but an educated mother is essential. A mother needs to have this quality. A mother must be educated. She needs to be smart. She needs to know whether the baby is being fed properly, cleaned properly, sent to school, and so forth … … ..See, now we have digital Bangladesh, if a mother is educated she will know what a baby needs …*. ; *what should be given to him or her and what shouldn’t. A mother can take care of all these things. Therefore, an educated mother is needed in every family …*” *FGD, Mothers, Basakarach, Derai.*

### Paternal capabilities

#### Being able to earn in all seasons and provide the family with the things they need

The fathers said they believe that if they are not able to provide essential items such as food, health care, clothes, and oil for hair and body massage, they are not taking good care of their wife and children. Several of the participants reported that they are under pressure to maintain a family with four to five children and other family members on a very tiny income ranging from 200 to 400 BDT (around $3 to $5) per day. They observed that if they had higher earnings, they might be better able to plan these essential purchases. The mothers in a FGD reflected on these challenges in the following way:

*“Number one they should work regularly, they must earn. If they earn 10 taka, they should save four taka for the children, one taka should be saved for health care for the children. The father must think about how he will get quality food for his children, good clothes for his children. Because the fathers are earning the mothers cannot earn.” FGD, Mothers, Basakarach, Derai.*

The main opportunities for earning in the region are associated with cultivating and harvesting rice during the dry season. As the local farmers have to rent land for cultivation, they are obliged to pay these rents with cash or a portion of their rice crop. If, however, there are unexpected hailstorms or early flood, the farmers may not be able to harvest their paddies and will suffer huge financial losses. According to the participants, other factors that affect the capability of men to earn and provide the family with essential items include limited job opportunities during the wet season, low wages, and low market prices for harvested rice.

#### Being able to save the future and the one who creates the future

The participants frequently mentioned that many mothers and new-born babies die in the *haor,* either because of a delay in reaching the health care centre, or because the care services they receive are of low quality. According to the fathers, one of the saddest experiences in life is losing the future they had planned and being unable to create another future. A father in a FGD expressed this experience in the following way:

*“See, before the marriage people dream of having a beautiful wife, then after getting married people dream of having babies. (Someone starts laughing). No do not laugh! Why do you laugh? When we talk about the important aspects of marriage you laugh! See these children are our future. Now to get this future, if the future along with the person through whom the future is delivered die, it takes us through the saddest experience of life. The saddest thing more than anything!” FGD, Fathers, Golbogi, Baniachang.*

The participants reported that most of the people living in the *haor* prefer to have a home delivery and tend to seek support from traditional birth attendants in order to avoid a caesarean delivery and to maintain their traditions. They said they believe that having surgery can interrupt women’s normal activities, as women need to be able to do heavy work. They admitted, however, that these birth attendants sometimes lack the skills to ensure a proper delivery. A father from *Baniachang* told the following story:

*“There was a dai (traditional birth attendant), while she was trying to do the delivery manually the baby got a stain on its head because of her hand, I saw it in my own eyes! Then the baby was delivered at Habiganj hospital, in town. After an hour the baby died. You see the stain on its head, that’s why!” FGD, Fathers, Keorakandi, Baniachang.*

The participants also reported that they are often unable to get medical care when needed because the facilities are too far away, or for a range of other reasons, such as a lack of money or of a vehicle, bumpy roads, windy and stormy weather, concerns about long travelling hours, or a doctor’s refusal to provide treatment at short notice. A mother in a FGD described her neighbour’s experience as follows:

*“She had bleeding during the delivery. When she was taken to the doctor, the doctor refused … … … .. They didn’t get the boat in time, she later died from persistent bleeding.” FGD, Mothers, Sarail, Baniachang.*

#### Being able to express love by purchasing toys and other items as required

The participants stated that most fathers have a limited ability to take care of their children because they work outside the home. Fathers are expected to provide their children with care and love by, for example, playing with them after returning from work, taking them on outings, bringing them medicine when they are sick, and bringing them toys or food. A father described how he shows his love for his baby:

*“Today I brought a toy car for the baby to play with. The baby doesn’t like the toy car, so I brought a jhunjhuni (a bell-like toy that makes sounds while shaking); the baby doesn’t like it so I brought toys like a duck. Whenever the baby wants any toy I bring it.” IDI, Father, Kadirpur, Derai.*

#### Being able to access the education needed to get a job and educate the children

Capabilities to be educated was observed as a need for a father in order to get a good job, educate his children, achieve a certain social status, and maintain his family. It was, for example, pointed out that an uneducated father can hardly understand the importance of education for his children. A mother described this capability in the following way:

“If a father is educated he will be able to get a job and buy things for the children. In the future he will be able to help his children to get education and a job.” *FGD, Mothers, Sarail, Baniachang.*

#### A father needs to be healthy, energetic, have a brain (intelligence), and be religious

The participants expressed the needs for a father to be healthy and energetic so he can work hard, earn money, and take care of other family members. In addition, they observed that a father has to be intelligent in order to manage the family well, and has to be religious in order to understand what each of his children needs. A father explained why fathers must have certain qualities:

*“First of all, a father needs to be healthy and energetic, otherwise he won’t be able to take care of his wife. Secondly, he needs to have a brain, or he won’t be able to maintain the family well. Thirdly, he needs to be religious, or he will not know which of his children needs what. A man should have these three qualities.” FGD, Fathers, Golbogi, Baniachang.*

### At the household level

#### Being able to live in a safe home during the wet season

The participants reported that the people living in the *haor* are constantly vigilant about protecting their homes from natural calamities such as sudden storms and high flood waters. They use specific bamboo materials sourced locally called *aar* to protect their houses from floodwaters. A father explained how the severe weather can endanger local homes:

“* …* *When the water overflows the area ... it ravages the houses. If a strong wind or a storm ravages the houses will it not create problems for you?” ID, Father, Chandipur, Baniachang.*

Many of the participants reported that they live in tiny houses that are almost falling apart. During the wet season, the yards around their houses are underwater, which requires them to pay extra attention to the children to save them from drowning.

#### Being able to overcome struggles with the earth to stay neat and clean

The participants reported that when the mothers, children, and fathers in their communities are all struggling against the forces of nature in order to survive, they hardly have the luxury to think about maintaining hygiene or staying clean using soap and oil. They explained that because their children grow up amid mud and dust, it is not feasible to keep them neat and clean. A father described these challenges in the following way:

*“We struggle with the earth every day. For example, my wife and children all fight with the earth every day. I have two children; they were just given shower with oil and water. After half an hour they will be covered in dust and mud. Now how many times you can shower your children?” FGD, Fathers, Golbogi, Baniachang.*

Some of the participants mentioned that only around 25% of the *haor* communities use soap for cleaning or after defecation (expressed as four *Anna* out of 16 *Anna)*. The key reasons given for not using soap were a lack of time, having no access to soap, being unwilling to clean, a lack of knowledge about hygiene, and having no access to a latrine. The mothers in a FGD explained how cleanliness is prioritised as follows:

*“There is hunger inside the stomach. The first priority is to satisfy that. Those who have many income sources, they can manage. They can get oil or soap.” FGD, Mothers, Basakarach, Derai.*

#### Being able to be mobile during different seasonal periods

The capability to be mobile and to maintain a normal life throughout the year appears to be a major challenge in the *haor* region. During the wet season, residents always need to hire a boat to engage in their daily activities, such as going out to work, accessing health care, and purchasing essentials. As hiring a boat can be expensive, the participants reported getting help from their neighbours by, for example, asking them to pick up some essential items when they are traveling somewhere. They noted that it is risky to travel by boat or by ferry when the weather turns stormy. Thus, in many cases, people cannot reach their destinations on time. The fathers in a FDG described these mobility challenges as follows:

*“... During the wet season, if the tide gets strong, it is not possible to operate or row the boat. That’s why people are late …*” *FGD, Fathers, Makhonia, Baniachang.*

The participants frequently mentioned that mobility for engaging daily activities or going shopping is easier during the dry season. However, they reported that they still face challenges when a person is sick and needs to be moved to get health care. They explained that in such cases they tend to use a hammock or a *thelagari* (a vehicle with four wheels that is pulled manually) to transport the patient to the health care centre in order to avoid the bumps in the rough roads that are especially problematic when using motor vehicles. The fathers in a FGD described the perils of moving sick people in the dry season:

*“... Now if half of us stay home we will remain well but if we move through the street ...it is very difficult … (interjection by another participant: The bumping of the vehicle makes the condition serious)!... If your disease is at 50% it will become 25% more severe. The condition will get worse...” FGD, Fathers, Golbogi, Baniachang.*

The participants also noted that mobility can be even more challenging during the seasonal transition phases, as the vehicles cannot run on the muddy tracks. In such circumstances, community members often band together to carry a patient to the hospital on their shoulders or in a hammock, which can take time. A mother told the story of how her neighbour was moved in a medical emergency:

“*… She was bleeding; she was not able to walk. Then we became confused what to do …*. *The water level had reached the lowest layer; it was neither increasing nor decreasing; neither a car nor a boat could be operated. In this case, four to five people used a hammock with a bamboo frame to carry the patient on their shoulders to Baniachang.” FGD, Mothers, Sarail, Baniachajng.*

#### Being able to access safe drinking water for the children and the family

The participants indicated that they value having access to safe drinking water for their children. It was pointed out that pond or river water is unsafe for drinking, though it is used for washing dishes, doing laundry, and showering animals. The participants observed that people develop diseases like diarrhoea and dysentery when they lack a source of clean drinking water. A mother described the dangers of drinking unclean water:

*“You see the pond here, people take water from this pond for both bathing and drinking. The livestock are bathed in the same water. Using this water makes people sick …*” *FGD, Mothers, Basakarach, Derai.*

The participants reported that it is difficult for them to get water from a tube well during the flooding period if it is located far from the house. Moreover, in the dry season, the tube wells are used for irrigation, which reduces the flow of available water. Other factors influence the participants’ capability to access safe water, including tube wells being located inside the mosque (where women are not allowed to go), an inadequate number of tube wells, and a lack of ownership of water sources. A father explained the problems his community faces in accessing water as follows:

*“See, we are 10 families here, but we don’t have a single tube well. A tube well for 100 families, does it sound okay? No, right?” FGD, Fathers, Alinagar, Derai.*

## Discussion

In this study, we applied a capability approach framework to explain the complexity of child growth in the context of the *haor* region. While the capability approach has been used as a normative framework in a wide range of areas of health and social science, its operationalisation and use in the analysis of empirical data in general, and of child growth in particular, has been limited [23–27]. Most of the empirical studies that tried to compile a list of capabilities were based on the available secondary data, a proxy list of central human capabilities published by Nussbaum, and normative assumptions [31–35]. Moreover, as Nussbaum’s list of central human capabilities was drawn up from the perspective of human rights, it doesn’t reflect the reasons why certain people value certain capabilities, and at which ages [36]. In this paper, the list was drawn up in discussion with the communities in the *haor* with a focus on child growth in the first thousand days of life.

The descriptions of and the reasoning underlying each of the capabilities presented indicate that the capabilities are interrelated, and thus that one type of capability creates space for shaping another capability for achieving healthy growth [22–24]. For example, if a child is free from disease, s/he eats, plays, and stays peaceful and joyous. Similarly, the parents’ capabilities to provide good care for their children and to achieve certain outcomes for their own wellbeing help to shape the additional capabilities of themselves and their children [23, 24]. For example, by eating well and staying healthy, a mother’s capability to provide good care for her children is enhanced, and the children become capable of being healthy, fed, loved, and cared for. A father’s capability to earn and meet his family’s essential needs enables the mother and the children in the family to access other items they need to survive and grow. Thus, application of CFCG sheds new lights to the existing frameworks on child growth, nutrition and social determinants of health, which traditionally assessed the multidimensional context at the *determinants* side [19, 20, 37]. In this study the interactions between these capabilities illustrate that child growth *outcomes are plural* and go beyond the biometric measures such as child survival, psychosocial development, health, and appropriate feeding to include the multidimensional achievements of parents.

The stories of the participants clearly show that they struggle to perform the most basic tasks needed to ensure the survival of themselves and their children. Because the region where they live suffers from long-term flooding, limited job opportunities, and agricultural losses, their freedom to provide their children with the basic items they need is severely constrained. The participants’ stories also indicate that few social safety nets are available to cushion their vulnerabilities during the wet season. Their capabilities to do something are contingent not only on seasonal conditions, but on what they want to achieve in which season. For example, the transportation infrastructure available during the dry season provides them with a sufficient level of mobility to go out to work, but it acts as a barrier when the goal is to take a patient to the health care centre. It appears that the participants sometimes lacked a capability in one season that they had in another because the conversion factors were different, or they lacked another capability or the required resources. For example, some people lacked the capability to access safe drinking water during the wet season and the transition phases because they lacked the capability to be mobile; whereas during the dry season they lacked access to safe drinking water because the water was being used for irrigation purposes. Some of the participants also described access to water as an availability issue that is socially embedded, and that can only be addressed when safe water is provided or available.

The reach and the meaning of each of the capabilities for the fathers and the mothers were reflected in their socially assigned roles and responsibilities. For example, the fathers were expected to play the role of breadwinner, whereas the mothers were expected to provide most of the child care. In much of the world, nurturing and caring for the family are seen as the responsibilities of women, while the responsibility to provide the family’s material resources is mainly assigned to men. In some contexts, the institutions controlling the rules, regulations, and societal structures are not gender-sensitive as they are grounded in socially ascribed obligations [38–40]. In context of the *haor* region, the mothers’ capabilities to practice autonomy in allocating time for child care, to become financially empowered, or to stand up to patriarchal violence are determined by their societal arrangements, including their household responsibilities, their access to women-friendly job opportunities, and their degree of conformity to patriarchal violence. The trade-off of time the mothers make in performing unpaid domestic chores and child care, their financial dependency on their husbands, and their mental wellbeing affect a range of other capabilities associated with the health and nutrition of the mothers and their children [41–44].

The study’s findings were restricted to the emic views of the participants. Therefore, the list of capabilities may include dimensions that are not aligned with the views of experts or the existing evidence. For example, the capability to be born with God’s blessing or to overcome the barriers to staying neat and clean may not be found in the experts’ lists or in the literature, but they came out in these stories. However, local perceptions and biomedical evidence might just use a different language. “Heredity” and “God’s will” as causes of stunting mentioned by our participants, could be interpreted in the light of the biomedical paradigm as (epi) genetic factors [10, 45, 46]. This is important when designing interventions: aligning public health messages with local perceptions and beliefs will contribute to an improved understanding between health professionals and the target population. The list may exclude dimensions that were not seen as important by the participants, but were considered important by the experts. For example, vaccination, family planning services, and indoor pollution might be factors that influence child growth outcomes, but they were not mentioned by the participants, and were therefore not included in the list. To minimise such limitations, the “emic” list can be further discussed with communities and experts across other regions of the country, which would allow for cross-boundary scrutiny of the list and the inclusion of an expert view [47].

## Conclusions

Our findings suggest that in *haor* areas, a multidimensional approach where the focus is not confined to the *determinants level* but expanded to the *aggregated growth* outcomes could further advance our efforts to improve child growth and achieve higher levels of child survival. The existing efforts define and assess child growth as an anthropometric outcome only, and include multisectoral interventions that focus on the distribution of resources and service coverage. These efforts need to be expanded based on an understanding of what parents are and are not capable of doing for their children, and why. As this study addressed these previously unanswered questions, our findings could play an instrumental role in the design of interventions aimed at expanding the multidimensional capabilities for child growth in the *haor.* The first steps in the design of interventions aimed at achieving healthy levels of child growth may include finding ways to enhance the identified capabilities of the *haor* communities in adverse circumstances. This can be done in two steps: first, by collecting insights from the existing programmes that are implementing interventions in *haor* areas on how these findings could be converted for the children in the *haor;* and second, from the stories of communities that were in similarly adverse situations but are now doing better and have achieved better outcomes than the communities of the *haor*. Finally, these two levels of insights can be translated into designing interventions that could be implemented in a range of communities with poor growth outcomes.

## Supplementary information

**Additional file 1. Interview Guides and Code Book.**

## Data Availability

We have shared the code book and interview guides as Additional file. The transcripts of this study are available in the native language of *haor* areas of Bangladesh and can be made available from the corresponding author based on request.

## Abbreviations

BMI: Body Mass Index
CA: Capability Approach
CFCG: Capability framework to child growth
FGD: Focus group discussions
IDI: In-depth interviews
MUAC: Mid Upper Arm Circumference
SGA: Small for gestational age
