# Supplementary material for: “We struggle with the earth everyday”: parents’ perspectives on the capabilities for healthy child growth in haor region of Bangladesh
Source: BMC Public Health. 2020 Jan 31;20:140. doi: 10.1186/s12889-020-8196-9 (PMC6993497; doi:10.1186/s12889-020-8196-9)
Supplement: Supplementary file 1 — Additional file 1. Interview Guides and Code Book. [file 12889_2020_8196_MOESM1_ESM.docx]

Additional file 1

**Contents**

[**Interview Guides-Indepth Interview (IDI)** 2](#_Toc523132003)

[**IDI for Mother (Consent Form in Bangla)** 2](#_Toc523132004)

[**IDI for Mother (Consent Form in English)** 3](#_Toc523132005)

[**IDI (Topic guide for the Mothers)** 4](#_Toc523132006)

[**IDI for Father (Consent Form in Bangla)** 10](#_Toc523132007)

[**IDI for Father (Consent Form in English)** 11](#_Toc523132008)

[**IDI (Topic Guide for Fathers)** 12](#_Toc523132009)

[**Interview Guides-Focus Group Discussions (FGD)** 18](#_Toc523132010)

[**FGD (Consent Form in Bangla)** 18](#_Toc523132011)

[**FGD (Consent Form in English)** 19](#_Toc523132012)

[**FGD Topic Guide** 20](#_Toc523132013)

# **Interview Guides-Indepth Interview (IDI)**

## **IDI for Mother (Consent Form in Bangla)**

**ZvwiL:**

**Introduction**

Avm&mvjvgyAvjvBKzg/bg®‹vi, Avgvi bvg.......................................................| Avwg Avcbvi Kv‡Q GKwU M‡elbvi Kv‡R G‡mwQ| G M‡elbv Kvh©µgwU †b`vij¨vÛ Gi Groningen University, eª¨vK evsjv‡`k Ges LANSA Gi †hŠ_ D‡Ï¨v‡M DFID Ges Eric Bleumink Fund (EBF) Gi mn‡hvMxZvq cwiPvwjZ n‡”Q**| G** M‡elbv Kvh©µ‡gi D‡Ïk¨ n‡jv nvIi GjvKvi wkï cywói wewfbœ fv‡jvg›` w`K¸‡jv wbY©q Kiv| GRb¨ Avwg Avcbvi wkï I Avvcbvi ¯^v¯’¨ I cywóMZ Ae¯’v m¤ú‡K© K_v ej‡ev|

mvÿvrKviwU m¤úbœ Ki‡Z 50-60 wgwbU mgq jvM‡Z cv‡i| Avcbvi m`q AbygwZ mv‡c‡ÿ G mvÿvrKviwU Avgiv wj‡L wbe Ges ‡iKW© Ki‡ev| G mvÿvrKv‡i AskMÖnb Kiv ev bv Kiv Avcbvi m¤ú~Y© B”Qvaxb| Avcwb ‡h‡Kvb mgq †Kvb Kvib cÖ`k©b QvovB G mvÿvrKvi eÜ Ki‡Z cv‡ib| Z_vwc Avcbvi gZvgZ I `„wófw½ Avgv‡`i Kv‡Q LyeB ¸iZ¡c~Y©| G‡ÿ‡Î mwVK ev fzj DË‡ii ‡Kvb welq †bB| Avcwb †Kvb cÖ‡kœi DËi w`‡Z ms‡KvP †eva Ki‡j Avgv‡K Rvbv‡Z cv‡ib Ges cÖ‡kœi DËi bv I w`‡Z cv‡ib|

AvR‡K Avgiv Avcbv‡`i †Kvb †mev ev civgk© ev Avw_©K myweav (UvKv-cqmv) w`‡Z Avwm wb, eis Avcbvi KvQ †_‡K nvI‡ii eZ©gvb Ae¯’v Ges G GjvKvi wkï‡`i ¯^v¯’¨MZ Ae¯’v m¤ú‡K© Rvb‡Z G‡mwQ| Avcbvi mvÿvrKvi †_‡K cÖvß Z_¨ Avcbv‡`i mvwe©K Ae¯’v eyS‡Z mvnvh¨ Ki‡e Ges Avcbv‡`i Ae¯’vi Dbœq‡bi j‡ÿ¨ KZ©„cÿ‡K Rvbv‡bvi †ÿ‡Î mvnvh¨ Ki‡e| G M‡elbvi djvdj ïaygvÎ ˆeÁvwbK Kv‡Ri Rb¨ cÖKvk Kiv n‡e Ges Avcbvi bvg †Kv_vI cÖKvk Kiv n‡e bv| mvÿvrKv‡i AskMÖn‡bi d‡j eª¨vK ev Ab¨ †Kvb msMVb †_‡K cÖvß †mevi Dci †Kvb Lvivc cÖfve co‡e bv| Avcwb wbwØ©avq G mvÿvrKv‡i AskMÖnb Ki‡Z cv‡ib| Avcbvi G AskMÖnb Avgv‡`i mK‡ji Kv‡Q cÖkswmZ n‡e| Avcwb wK mvÿvrKv‡i AskMÖnb Ki‡Z AvMÖnx ?

**1= n¨uv 2= bv**

AskMÖnbKvix ivRx n‡j Zvi ¯^vÿi wb‡q mvÿvrKvi ïiæ Kiæb|

**AskMÖnbKvixi bvg:**

**AskMÖnbKvixi ¯^vÿi:**

**mvÿvrKvi MÖnbKvixi bvg: ¯^vÿi I ZvwiL:**

## **IDI for Mother (Consent Form in English)**

**Date:**

**Introduction**

Hello, my name is ……………………………………………………….. I came to you to conduct a research project that is jointly initiated by the BRAC, Bangladesh, Groningen University of the Netherlands and LANSA funded by DFID and Eric Bleumink Fund (EBF). The main objective of the research project is to understand the issues regarding child nutrition, good or bad, in *haor* areas. For this I would like to talk with you about you and your child’s health and nutritional condition.

The interview may take around 50-60 minutes. In conducting the interview, we would like to take notes and record your responses if you kindly allow. Your participation is completely voluntary and you may withdraw from the study at any point without giving any reason. However, we value your opinion and views that you will share with us. There is no right or wrong answer. If you feel uncomfortable with any question, you can let us know and can choose not to answer that question.

We haven’t come to you in providing any service or advice or money, rather we came here to know from you about the scenario in *haor*. Your participation in the research will help us understand the situation and communicate it to relevant stakeholder to improve their efforts. The findings of this research would be published in scientific journal and your name will be kept anonymous. It will bring no consequence to the services that you receive from BRAC or others. So you can be comfortable to answer as you feel. We would appreciate if you give us your valuable time for this interview. Do you agree?

**1= Yes 2= No**

If the participant agrees take his/her signature and continue with the interview:

**Name of the Participant: Signature of the Participant:**

**Interviewed by: signature and Date:**

## **IDI (Topic guide for the Mothers)**

**Background Information:**

**ID No: …………………….. Date:**

**AskMÖnbKvixi eqm (Age of the participant):**

**mšÍv‡bi msL¨vÑRxweZ I g„Z (Number of children, alive and lost):**

**2 eQ‡ii Kg eqmx mšÍv‡bi msL¨v (Number of u-2 children):**

**me †_‡K ‡QvU mšÍv‡bi eqm (Age of the youngest child):**

**Lvbvi m`m¨ msL¨v (Number of people in the household):**

**ag© (Religion):**

**Dc‡Rjv (Upazilla):**

**MÖvg (Village):**

**AskMÖnbKvixi wkÿvMZ Ae¯’v (Education of the participant):**

**AskMÖnbKvixi †ckv (Occupation of the participant):**

**Lvbv cÖav‡bi bvg (Household head’s name):**

**Lvbv cÖav‡bi mv‡_ m¤úK© (Relationship with household head):**

**AskMÖnbKvixi ¯^vgxi wkÿvMZ Ae¯’v (Education of husband):**

**AskMÖnbKvixi ¯^vgxi †ckv (Occupation of husband):**

**IDI Questions**

**Opening questions:**

1. Avcbvi mv‡_ memgq hviv _v‡Kb Zv‡`i m¤ú‡K© GKUz e‡jb| (wRÁvmv Kiæb**:** ¯^vgx, evev-gv, kïo-kvïox, †Q‡j‡g‡q BZ¨vw`)|

Would you tell me about the people you live with? (Probes: Husband, parents, in-laws, number of children etc.)

1. mvaviYZ cÖwZw`b Avcwb wK KvR K‡ib? cÖwZw`‡bi Kv‡R †K Avcbv‡K me‡P‡q †ekx/memgq mvnvh¨ K‡i?

What do you usually do every day? Who else mostly cooperate or constantly accompany you while doing your daily work?

1. Avcbvi Lvbvq, ¸iZ¡c~Y© wm×všÍ¸‡jv mvavibZ ‡K MÖnb K‡ib? (wRÁvmv Kiƒb: msmv‡ii cÖ‡qvRbxq wRwbmcÎ †Kbv, mšÍvb‡`i cov‡kvbv, Lvevi: Lvevi †Kbv, wK ivbœv n‡e, †K wK Lv‡e, ¯^v¯’¨‡mev/wPwKrmv wel‡q|)

Who usually take the key decisions in your household? (Probes: Purchasing of household items, education of the children, food: purchasing, consumption, menu selection, health care etc)

**Seasonality agriculture and income:**

Avcbvi GjvKvq wewfbœ mg‡q †h eb¨v nq Avevi wKQzw`b ïKbv _v‡K †m m¤ú‡K© wK Avgv‡K wKQz ej‡eb? (wRÁvmv Kiƒb: KLb nq, KLb ïiæ nq, KZ mgq a‡i GUv _v‡K, KLb †kl nq, Gi d‡j Zvi cwievi wKfv‡e cÖfvweZ nq )

Would you tell me about the scenario of flood, drought in your area? (Probes: what happens, when it starts, how long it persists, when it recedes, its impact on you and your family)

mvavibZ Avq †ivRMvi/iæwR Kivi Rb¨ Avcwb wK K‡ib? †Kb Avcwb G KvR K‡ib? (wRÁvmv Kiæb: ïKbvi mgq, eb¨vi mgq, gvSvgvwS mgq) KLb/†Kvb mgq Avcbvi ‡ivRMvi/iæwR fvj nq? ‡Kb? ‡Kvb mgqUv Avcbvi Lvivc hvq? †Kb? hLb GB mgm¨v¸‡jv nq Avcwb wK K‡ib? Avcwb ‡h UvKv †ivRMvi/iæwR K‡ib Zv Li‡Pi e¨vcv‡i †K wm×všÍ †bq? †Kb?

What do you usually do for your livelihood and why? (Probes: dry season, flood period, transition period) In your case when do you earn better and why? When do you feel worse and why? How do you manage when problem arises? Who usually decides how the money you earn will be used? Why?

1. Avcwb †h KvR ¸‡jvi K_v ej‡jb †m¸‡jv QvovI Avcbvi cwiev‡ii Ab¨vb¨ m`m¨iv wK KvR K‡i? (wRÁvmv Kiƒb: †K wK KvR K‡i) †Kb? (wRÁvmv Kiƒb: Pvlvev`/avb Pvl, evwoi mvg‡b dj I kvK-me&wRi evMvb, nuvm gyiMx cvjb, grm Pvl I Ab¨vb¨ KvR m¤ú‡K© †hgb: †bŠKv Pvjv‡bv)

In addition to the above (as mentioned by the participant) what else is done by your household members (ask by whom) and why? (Probes: rice cultivation, vegetables or fruits gardening, poultry or livestock rearing, or fishing or any other activities such as boating)

7. eb¨vi mgq wKfv‡e G KvR¸‡jv K‡ib? (wRÁvmv Kiæb: Pvlvev`/avb Pvl, evwoi mvg‡b dj I kvK-me&wRi evMvb, nuvm gyiMx cvjb BZ¨vw` Kv‡Ri †ÿ‡Î) ïKbvi mgq G KvR¸‡jv K‡ib? hLb cvwb ïKv‡Z _v‡K ZLb wK nq? G iKg K‡e n‡qwQj? ZLb wK K‡iwQ‡jb? (wRÁvmv Kiæb: wKfv‡e cwiKíbv K‡iwQj, wK Pvl K‡iwQj, wKfv‡e exR evQvB K‡iwQj, wKfv‡e Rwg Pvl K‡iwQj, wK iKg djb n‡qwQj, weµq Kiv‡Z †c‡iwQj wKbv? bv cvi‡j ‡Kb cv‡iwb BZ¨vw` m¤ú‡K©)

How do you plan these activities (that we just talked about for example, rice cultivation or gardening, or livestock/poultry rearing etc) given the likelihood during floods. How do you plan these activities in dry season? What happened during transition period? Would you share any relevant experience in this regard? (For agriculture, probes: planning, selection of seeds, cultivation, production and market opportunity)

Avcwb wK Avgv‡K ej‡eb †h Avcwb K…wli gva¨‡g hv hv Drcv`b K‡ib †m¸‡jv w`‡q wK K‡ib/wK Kv‡R jv‡M? (wRÁvmv Kiæb: LvIqv:wK cwigvb Lvq, wK cwigvb Lvq, KZUzKz mvkÖq nq; wewµ Kiv: wewµ Ki‡Z cv‡i wKbv, Avw_©K jvf/ÿwZ) mvavibZ (K„wlKvR ev dm‡ji †ÿ‡Î) Avcbvi wK ai‡bi ÿwZ n‡q _v‡K? K‡e, wKfv‡e Avcbvi G iKg ÿwZ n‡qwQj? (wRÁvmv Kiæb: Avw_©K fv‡e wKfv‡e ÿwZ n‡qwQj, wK cwigvb dmj bó n‡qwQj Ges wKfv‡e, Drcv`b Kg n‡qwQj wKbv, wewµ Ki‡Z Amyweav n‡qwQj wKbv, BZ¨vw` m¤ú‡K©) ZLb Avcwb wK K‡iwQ‡jb (wKfv‡e ÿwZ c~iY K‡iwQ‡jb/ wKfv‡e mvgvj w`‡q‡Qb)?

How do you get benefitted receive through your farming production? (Probes: consumption: how much is consumed, surplus, how the surplus is used market opportunity) What kind of loss do you face usually? Would you share any such experience that you had? How did you mitigate it? (Probes: financial, spoiled, flooded, limited market opportunity, didn’t grow well etc.)

AskMÖnbKvix K…wlKv‡Ri cvkvcvwk Ab¨ †Kvb Kv‡Ri K_v D‡jøL K‡i _vK‡j (†hgb: †bŠKv Pvjv‡bv) GKB fv‡e cÖkœ¸‡jv Kiæb| ‡hgb: (AskMÖnbKvixi D‡jøwLZ) G KvR/¸‡jv K‡i wKfv‡e Avcbvi DcKvi nq? G wel‡q Avcbvi AwfÁZv m¤ú‡K© wKQz e‡jb| (wRÁvmv Kiæb: KvRwU Kivi myweav/Amyweav, Avw_©K jvf/ÿwZ, wKfv‡e ÿwZ c~iY K‡ib BZ¨vw` wel‡q)

If the participant mentions about boating or other activities in addition to farming, ask in the same way, how does it (as mentioned by participant) benefit you? Would you share any example? (Probes: benefits/difficulties, profits/loses, how to mitigate etc)

**Perception on child caring: growth and nutrition:**

**Child Growth:**

1. Avwg GLb Avcbvi mšÍvb‡`i wel‡q K_v ej‡ev| Zviv †Kgb Av‡Q? Avcbvi me ‡_‡K †QvU mšÍvb m¤ú‡K© wKQz e‡jb| Zvi bvg wK? mvaviYZ †K Zvi (ev”Pvi bvg) †`Lv‡kvbv/ cwiPh©v K‡i? wKfv‡e? Zvi (ev”Pvi bvg) †`Lv‡kvbv Kivi Rb¨ Avcwb wK wK K‡ib? hLb Avcbvi N‡ii KvR K…wlKvR _v‡K ZLb wKfv‡e Zvi (ev”Pvi bvg) †`Lv‡kvbv/ hZœ K‡ib? †h Zvi †`Lv‡kvbv/ cwiPh©v Kivi Rb¨ †K me ‡_‡K †ekx Avcbv‡K mvnvh¨ K‡i? wKfv‡e?

Now let’s talk about your children. How are they doing? Can you talk a bit about the youngest child (ren)? What's his/her name(s)? Who usually takes care of [NAME] and how? Can you tell about your own role for his/her caring? How do you manage to take care of your child when you are to do other household works or agricultural activities or other works? Who mostly helps you in taking care of the child and how?

1. GKRb fvj evev nIqvi Rb¨ wK wK ¸b _vKv `iKvi e‡j g‡b K‡ib? (AskMÖnbKvix hZ¸‡jv welq‡K ¸iZ¡c~Y© g‡b K‡ib, me¸‡jv welq‡K we‡ePbvq Avb‡Z n‡e|) Avcbvi (ev”Pvi evevi) wK Ae¯’v?

What kind of qualities/capacities does a father need to have in taking care of his child (Probes: Consider all the important things that the participant consider important). In your case what happens?

1. GKRb fvj gv nIqvi Rb¨ wK wK ¸b _vKv cÖ‡qvRb/`iKvi e‡j g‡b K‡ib? (AskMÖnbKvix hZ¸‡jv welq‡K ¸iZ¡c~Y© g‡b K‡ib, me¸‡jv welq‡K we‡ePbvq Avb‡Z n‡e) Avcbvi wK Ae¯’v?

What kind of qualities/capacities does a mother need to have in taking care of her child (Probes: Consider all the important things that the participant consider important). In your case what happens?

1. Avcbvi wkïi †ÿ‡Î Avcwb wKfv‡e eyS‡Z cv‡ib †h Avcbvi ev”Pv wVKg‡Zv eo wKbv? wK †`‡L/wKfv‡e eyS‡Z cv‡ib †h Avcbvi ev”Pv wVKg‡Zv eo n‡”Q? (wRÁvmv Kiæb: wkï‡K Lvevi,¯^v¯’¨, cwi®‹vi-cwi”QbœZv, ‡Ljva~jv Kiv, Ab¨‡`i mv‡_ †g‡k wKbv A_ev Ab¨ †Kvb welq ‡h¸‡jv AskMÖnbKvix ¸iZ¡c~Y© g‡b K‡ib)

In your case how/when do you feel that your child is growing well or not? What do you do in making sure that your child is growing well? (Probes: food, health, sanitation, hygiene, playfulness, social interaction, or any other contextual issues as considered important by the participant)

1. KLbI wK Ggb n‡qwQj †h Avcbvi ev”Pv wVKg‡Zv eo nw”Qj bv? Avcwb wKfv‡e eyS‡Z ‡c‡iwQ‡jb? (wRÁvmv Kiæb e„w× bv nIqvi wewfbœ kvwiixK jÿb: Amy¯’ nIqv, D”PZv, IRb, Mv‡qi is, Pz‡ji is BZ¨vw`, mvgvwRK I Ab¨vb¨ welq: ‡Ljva~jv bv Kiv, Ab¨‡`i mv‡_ bv †gkv, wLUwL‡U †gRvR BZ¨vw` mn †hme welq AskMÖnbKvix ¸iZ¡c~Y© e‡j g‡b K‡ib) Avgv‡K wK GKUz ej‡eb †h ZLb wK n‡qwQj? wK Kvi‡Y G iKg n‡qwQj e‡j Avcbvi g‡b nq? Avcbvi g‡Z wK Ki‡j G ai‡bi mgm¨v n‡e bv?

Have any of your child ever experienced poor growth? How did you know? (Probes: markers of poor growth i.e. biomedical: illness, height, weight, skin, hair; non-biomedical: playfulness, social interaction, irritability other contextual issues as considered important by the participant). Would you tell the story what happened? What might be the possible reasons for that? How such problem (poor growth) could be prevented?

1. wKQz wKQz wkï‡K j¤^vq Kg/Lv‡Uv g‡b nq| G welqwU‡K Avcwb wKfv‡e †`‡Lb? †Kb GiKg nq? (G wel‡q Zvi fv‡jv ev g›` Abyf‚wZi K_v wRÁvmv Kiæb)

There are some children who are considered to be stunted. What do you think, why it happens?

1. Avevi wKQz wkï‡K ¯^v¯’¨evb ev ‡gvUv‡mvUv g‡b nq| G welqwU‡K Avcwb wKfv‡e †`‡Lb? †Kb? Avevi wKQz ev”Pv LyeB †ivMv-cvZjv nq| G welqwU‡K Avcwb wKfv‡e †`‡Lb? †Kb? (G wel‡q Zvi fv‡jv ev g›` Abyf‚wZi K_v wRÁvmv Kiæb)

Some children are also considered to be fat. How do you see that? Why? On the other hand some children are also considered to be thin. How do you see that? Why? (Probes: good, bad or as you feel)

**Child Nutrition, Health and Interventions:**

1. Avwg GLb ev”Pv‡`i LvIqv-`vIqv I Zv‡`i cywó wel‡q K_v ej‡ev| Avcbvi ev”Pv‡K mvavibZ †K LvIqvq Ges †Kb? mvavibZ Avcbvi ev”Pv‡K wK wK (Lvevi) LvIqv‡bv nq/LIqvb? (wRÁvmv Kiæb: ey‡Ki `ya LvIqv‡bv: KZw`b ïaygvÎ ey‡Ki `ya LvB‡q‡Qb, KZw`b ey‡Ki `ya LvIqv‡eb, evowZ Lvevi LvIqv‡bv: K‡e ïiæ K‡i‡Qb/ Ki‡eb, wK ai‡bi Lvevi †`b, KZevi †`b, Lvev‡i ˆewPÎ¨ m¤ú‡K©)

Now I would like to talk a bit about your child’s feeding and nutrition. Would you tell who usually feed your child and why? Can you tell about the food that are usually offered/feed to your child? (Probes: breastfeeding: initiation, colostrum, duration of exclusive breastfeeding, continuation of breastfeeding, complementary feeding: initiation, type of foods provided, frequency, variation in diets etc., food taboos)

1. Avcbvi ev”Pvi fv‡jv ¯^v¯’¨ I cywói Rb¨ Avcwb mvavibZ wK wK K‡ib ev wK ai‡bi evowZ hZœ †bb? (wRÁvmv Kiæb: cwi¯‹vi-cwi”QbœZv, LvIqv‡bvi Av‡M, b¨vcwKb e`jv‡bvi c‡i, gjg~Î Z¨v‡Mi ci ev”Pv‡K cwi®‹vi Kivi ci nvZ †avqvi Af¨vm m¤ú‡K©, Lvevi wbe©vPb, km¨ Drcv`b, †Ljva~jv, Ab¨‡`i mv‡_ †gj‡gkv Kiv)

What do you usually do/what extra cares do you provide to improve your child’s nutritional status? Probes: cleanliness: hand washing behavior before feeding, during changing of napkins, after defecation, selection of foods, investment for farming production, playing, hanging out, social interaction etc)

1. Avcwb Avcbvi ev”Pv‡K ‡hme Lvevi †L‡Z †`b †m¸‡jv wKfv‡e †hvMvo K‡ib? (wRÁvmv Kiæb: wK‡b Av‡b bvwK Drcv`b K‡i) eb¨vi mgq wK K‡ib? ïKbvi mgq wK K‡ib? hLb cvwb ïKv‡Z _v‡K H mgqUv‡Z wK K‡ib? (eb¨v/ïKbvi mgq wK ai‡bi Lvevi LvIqv‡Z cv‡ib, wKfv‡e †hvMvo K‡ib, wK ai‡bi Amyweavq ci‡Z nq BZ¨vw` wel‡q wRÁvmv Kiæb) G wel‡q Avcbvi AwfÁZv m¤ú‡K© GKUz e‡jb|

How do you get the foods that you offer to your child? (Probes: purchasing, agricultural production) What happens when there is flood? What do you do when the flood recedes or drought arises? (Usually which food are feed during flood/dry season, how they get those food, problems regarding this issue) Would you share any experience in this regard?

1. Avcbvi Gi kixi ¯^v¯’¨ †Kgb Av‡Q? †klevi hLb Avcbvi †c‡U ev”Pv wQj ZLb wK cwigvb/ wK ai‡bi Lvevi †L‡qwQ‡jb? H mgq Avcwb wK ‡Kvb evowZ hZœ †c‡qwQ‡jb? wKfv‡e? (wRÁvmv Kiæb: Lvevi, ¯^v¯’¨‡mev, cwiev‡ii hZœ, Kv‡Ri Pvc wel‡q)

How is your health now? Would you tell how much or what type of food you used to take during your last (Name) pregnancy? What kind of extra care were you given during that period? And How? (Probes: Diet, health care, family care, work load).

1. GLb wK Ae¯’v? GLb wK Avcbvi cwievi wKfv‡e Avcbvi hZœ †bq? Avcbvi ¯^vgx/kïo-kvïox Avcwb wKfv‡e Avcbvi ‡`Lv‡kvbv K‡i/hZœ? Avcwb wb‡Ri hZœ wKfv‡e †bb? †Kb? (wRÁvmv Kiæb: Lvevi, ¯^v¯’¨‡mev, cwiev‡ii hZœ, Kv‡Ri Pvc wel‡q)

What about now? How do the family care you? What about your husband and in-laws? How do you care on your own? Why? (Probes: Diet, health care, family care, work load)

1. Avcbvi g‡Z GKRb gv wKfv‡e wkïi ¯^v¯’¨ I cywói †ÿ‡Î f~wgKv cvjb Ki‡Z cv‡i? Avcwb wKfv‡e K‡ib?

Would you tell how a mother can contribute in enhancing child’s health and nutrition? In your case what happens?

1. GLb Avgiv ev”Pvi evevi wel‡q K_v ej‡ev| Avcbvi g‡Z GKRb evev wKfv‡e wkïi ¯^v¯’¨ I cywói †ÿ‡Î f~wgKv cvjb Ki‡Z cv‡i? Avcbvi ev”Pvi evev wK K‡ib? †m Avcbvi mv‡_ †Kgb AvPiY/e¨envi K‡i? †Kb? (wRÁvmv Kiæb: Mv‡q nvZ †Zvjv/AvNvZ Kiv, MvjvMvwj †`Iqv) G ai‡bi †Kvb NUbvi K_v wK Avgv‡K Rvbv‡eb? hLb G iKg n‡qwQj ZLb Avcbvi ev”Pvi wK †Kvb Amyweav n‡qwQj? (wRÁvmv Kiæb: ev”Pvi hZœ wb‡Z Amyweav, gv I wkïi gvbwmK Ae¯’v m¤ú‡K©)

Now let’s talk about the child’s father. In your opinion how a father can contribute in enhancing child’s health and nutrition? In your case what happens? How does he treat you? Why? (Probes: beating/hitting, abusing etc.). Would you share any story in this regard? How did it affect your baby? (Probes: problems in child caring, emotional both for mother and child etc.)

1. Avgiv GLb Avcbvi ev”Pvi ¯^v¯’¨ wel‡q K_v ej‡ev| Avcbvi ev”Pvi kixi ¯^v¯’¨ †Kgb Av‡Q? (wRÁvmv Kiæb: K‡e †m Amy¯’ n‡qwQj, wK n‡qwQj, †Kb) †m Amy¯’ n‡j wK K‡ib? eb¨vi mgq wK K‡ib? ïKbvi mgq ev”Pv Amy¯’ n‡j wK K‡ib? wKfv‡e mgm¨vi mgvavb K‡ib? hLb eb¨vi cvwb ïKv‡Z _v‡K (Pviw`‡K Kv`v _v‡K) †m mgq ev”Pv Amy¯’ n‡j wK K‡ib? Avcbv‡`i †ÿ‡Î KLbI Ggb n‡qwQj? ZLb wK K‡iwQ‡jb?

Let’s talk about your child health. How is his/her health now? (Probes: Did he suffer from any illness, when and why) What did you do for his/her recovery/what do you do when he/she gets sick? What happens when there is flood? How do you solve? And what happens during the transition phase? What about drought? Would share any of your experience in this regard?

1. hLb Avcbvi ¯^v¯’¨‡mevi cÖ‡qvRb nq ZLb wK K‡ib? (ev”Pvi bvg) hLb †c‡U wQj ZLb wKfv‡e ¯^v¯’¨‡mev wb‡qwQ‡jb? Zvi R‡b¥i mgq wK K‡iwQ‡jb? (†K ¯^v¯’¨‡mev w`‡qwQj, †Kv_vq, †Kb, wPwKrmvi gvb: e¨envi/AvPib, ch©vß ¯’vb, Av‡M †_‡K Rvbv‡Z nq wK bv, mš‘ó wKbv) eb¨vi mgq wK K‡ib? ïKbvi mgq wKfv‡e wPwKrmv/‡mev ‡bb? gvSvgvwS mg‡q wK nq? G iKg ‡Kvb NUbv wK n‡qwQj? †m m¤ú‡K© Avgv‡K wKQz e‡jb?

What do you do when you or the mother needs health care? What did you do during her pregnancy and delivery for (NAME)? (Probes: who provided the care, from where, why, quality of care: behavior, appointment issue, satisfaction) How do you manage when there is flood or drought arises or during the transition phase of flood and drought. Would you tell any relevant experience in this regard?

1. hw` Avcbvi wkï ev Lvbv m`m¨ †Kvb miKvix/‡emiKvix cÖwZôvb †_‡K mvnvh¨/mn‡hvMxZv/‡mev †c‡q _v‡Kb, †m m¤ú‡K© wK ej‡eb? (wRÁvmv Kiƒb: ¯^v¯’¨, cwi®‹vi-cwi”QbœZv, K…wl, Avw_©K wel‡q cÖvß mn‡hvMxZv/‡mev m¤ú‡K©) G ‡mevi gva¨‡g wKfv‡e Avcbvi DcKvi n‡q‡Q? wK ai‡bi mn‡hvMxZv/‡mev ‡c‡j Avcbvi AviI †ekx DcKvi n‡Zv e‡j Avcwb g‡b K‡ib?

Would you name the organizations from where you receive any kind of service or help? (probes: GO/NGOs/BRAC)? Would you tell who does what for you? What kind of services are particularly available for mother and child? (Probes: health, sanitation, hygiene, agriculture, financial) How useful are they? What could have been better?

Avgiv mvÿvrKv‡ii G‡Kev‡i †kl ch©v‡q P‡j G‡mwQ| †kl Kivi Av‡M Avcwb wK AviI wKQz ej‡Z Pvb? mvÿvrKv‡i AskMÖnb K‡i Avcbvi g~j¨evb gZvgZ cÖ`v‡bi Rb¨ ab¨ev` RvbvB|

We already came to the end of the interview, would like to add any other point before we conclude the discussion? Thanks for your valuable inputs and participation.

## **IDI for Father (Consent Form in Bangla)**

**ZvwiL:**

**Introduction**

Avm&mvjvgyAvjvBKzg/bg®‹vi, Avgvi bvg.......................................................| Avwg Avcbvi Kv‡Q GKwU M‡elbvi Kv‡R G‡mwQ| G M‡elbv Kvh©µgwU †b`vij¨vÛ Gi Groningen University, eª¨vK evsjv‡`k Ges LANSA Gi †hŠ_ D‡Ï¨v‡M DFID Ges Eric Bleumink Fund (EBF) Gi mn‡hvMxZvq cwiPvwjZ n‡”Q**| G** M‡elbv Kvh©µ‡gi D‡Ïk¨ n‡jv nvIi GjvKvi wkï cywói wewfbœ fv‡jvg›` w`K¸‡jv wbY©q Kiv| GRb¨ Avwg Avcbvi wkï I Avvcbvi ¯^v¯’¨ I cywóMZ Ae¯’v m¤ú‡K© K_v ej‡ev|

mvÿvrKviwU m¤úbœ Ki‡Z 50-60 wgwbU mgq jvM‡Z cv‡i| Avcbvi m`q AbygwZ mv‡c‡ÿ G mvÿvrKviwU Avgiv wj‡L wbe Ges ‡iKW© Ki‡ev| G mvÿvrKv‡i AskMÖnb Kiv ev bv Kiv Avcbvi m¤ú~Y© B”Qvaxb| Avcwb ‡h‡Kvb mgq †Kvb Kvib cÖ`k©b QvovB G mvÿvrKvi eÜ Ki‡Z cv‡ib| Z_vwc Avcbvi gZvgZ I `„wófw½ Avgv‡`i Kv‡Q LyeB ¸iZ¡c~Y©| G‡ÿ‡Î mwVK ev fzj DË‡ii ‡Kvb welq †bB| Avcwb †Kvb cÖ‡kœi DËi w`‡Z ms‡KvP †eva Ki‡j Avgv‡K Rvbv‡Z cv‡ib Ges cÖ‡kœi DËi bv I w`‡Z cv‡ib|

AvR‡K Avgiv Avcbv‡`i †Kvb †mev ev civgk© ev Avw_©K myweav (UvKv-cqmv) w`‡Z Avwm wb, eis eis Avcbvi KvQ †_‡K nvI‡ii eZ©gvb Ae¯’v Ges G GjvKvi wkï‡`i ¯^v¯’¨MZ Ae¯’v m¤ú‡K© Rvb‡Z G‡mwQ| Z‡e Avcbvi mvÿvrKvi †_‡K cÖvß Z_¨ Avcbv‡`i mvwe©K Ae¯’v eyS‡Z mvnvh¨ Ki‡e Ges Avcbv‡`i Ae¯’vi Dbœq‡bi j‡ÿ¨ KZ©„cÿ‡K Rvbv‡bvi †ÿ‡Î mvnvh¨ Ki‡e| G M‡elbvi djvdj ïaygvÎ ˆeÁvwbK Kv‡Ri Rb¨ cÖKvk Kiv n‡e Ges Avcbvi bvg †Kv_vI cÖKvk Kiv n‡e bv| mvÿvrKv‡i AskMÖn‡bi d‡j eª¨vK ev Ab¨ †Kvb msMVb †_‡K cÖvß †mevi Dci †Kvb Lvivc cÖfve co‡e bv| Avcwb wbwØ©avq G mvÿvrKv‡i AskMÖnb Ki‡Z cv‡ib| Avcbvi G AskMÖnb Avgv‡`i mK‡ji Kv‡Q cÖkswmZ n‡e| Avcwb wK mvÿvrKv‡i AskMÖnb Ki‡Z AvMÖnx ?

**1= n¨uv 2= bv**

AskMÖnbKvix ivRx n‡j Zvi ¯^vÿi wb‡q mvÿvrKvi ïiæ Kiæb|

**AskMÖnbKvixi bvg:**

**AskMÖnbKvixi ¯^vÿi:**

**mvÿvrKvi MÖnbKvixi bvg: ¯^vÿi I ZvwiL:**

## **IDI for Father (Consent Form in English)**

**Date:**

**Introduction**

Hello, my name is ……………………………………………………….. I came to you to conduct a research project that is jointly initiated by the BRAC, Bangladesh, Groningen University of the Netherlands and LANSA funded by DFID and Eric Bleumink Fund (EBF). The main objective of the research project is to understand the issues regarding child nutrition, good or bad, in *haor* areas. For this I would like to talk with you about you and your child’s health and nutritional condition.

The interview may take around 50-60 minutes. In conducting the interview, we would like to take notes and record your responses if you kindly allow. Your participation is completely voluntary and you may withdraw from the study at any point without giving any reason. However, we value your opinion and views that you will share with us. There is no right or wrong answer. If you feel uncomfortable with any question, you can let us know and can choose not to answer that question.

We haven’t come to you in providing any service or advice or money, rather we came here to know from you about the scenario in *haor*. However your participation in the research will help us understand the situation and communicate it to relevant stakeholder to improve their efforts. The findings of this research would be published in scientific journal and your name will be kept anonymous. It will bring no consequence to the services that you receive from BRAC or others. So you can be comfortable to answer as you feel. We would appreciate if you give us your valuable time for this interview. Do you agree?

**1= Yes 2= No**

If the participant agrees take his/her signature and continue with the interview:

**Name of the Participant: Signature of the Participant:**

**Interviewed by: signature and Date:**

## **IDI (Topic Guide for Fathers)**

**Background Information:**

**ID No: …………………….. Date:**

**AskMÖnbKvixi eqm (Age of the participant):**

**mšÍv‡bi msL¨vÑRxweZ I g„Z(Number of children, alive and lost):**

**2 eQ‡ii Kg eqmx mšÍv‡bi msL¨v (Number of u-2 children):**

**me †_‡K ‡QvU mšÍv‡bi eqm (Age of the youngest child):**

**Lvbvi m`m¨ msL¨v (Number of people in the household):**

**ag© (Religion):**

**Dc‡Rjv (Upazilla):**

**MÖvg (Village):**

**AskMÖnbKvixi wkÿvMZ Ae¯’v (Education of the participant):**

**AskMÖnbKvixi †ckv (Occupation of the participant):**

**Lvbv cÖav‡bi bvg (Household head’s name):**

**Lvbv cÖav‡bi mv‡_ m¤úK© (Relationship with household head):**

**AskMÖnbKvixi ¯¿x wkÿvMZ Ae¯’v (Education of wife):**

**AskMÖnbKvixi ¯¿x †ckv (Occupation of wife):**

**Opening questions:**

1. Avcbvi mv‡_ memgq hviv _v‡Kb Zv‡`i m¤ú‡K© GKUz e‡jb| (wRÁvmv Kiæb**:** ¯^vgx, evev-gv, kïo-kvïox, †Q‡j‡g‡q BZ¨vw`)|

Would you tell me about the people you live with? (Probes: Husband, parents, in-laws, number of children etc.)

1. mvaviYZ cÖwZw`b Avcwb wK KvR K‡ib? cÖwZw`‡bi Kv‡R †K Avcbv‡K me‡P‡q †ekx/memgq mvnvh¨ K‡i?

What do you usually do every day? Who else mostly cooperate or constantly accompany you while doing your daily work?

1. Avcbvi Lvbvq, ¸iZ¡c~Y© wm×všÍ¸‡jv mvavibZ ‡K MÖnb K‡ib? (wRÁvmv Kiƒb: msmv‡ii cÖ‡qvRbxq wRwbmcÎ †Kbv, mšÍvb‡`i cov‡kvbv, Lvevi: Lvevi †Kbv, wK ivbœv n‡e, †K wK Lv‡e, ¯^v¯’¨‡mev/wPwKrmv wel‡q|)

Who usually take the key decisions in your household? (Probes: Purchasing of household items, education of the children, food: purchasing, consumption, menu selection, health care etc)

**Seasonality agriculture and income:**

Avcbvi GjvKvq wewfbœ mg‡q †h eb¨v nq, Avevi wKQzw`b ïKbv _v‡K †m m¤ú‡K© wK Avgv‡K wKQz ej‡eb? (wRÁvmv Kiƒb: KLb nq, KLb ïiæ nq, KZ mgq a‡i GUv _v‡K, KLb †kl nq, Gi d‡j Zvi cwievi wKfv‡e cÖfvweZ nq )

Would you tell me about the scenario of flood, drought in your area? (Probes: what happens, when it starts, how long it persists, when it recedes, its impact on you and your family)

mvavibZ Avq †ivRMvi/iæwR Kivi Rb¨ Avcwb wK K‡ib? †Kb Avcwb G KvR K‡ib? (wRÁvmv Kiæb: ïKbvi mgq, eb¨vi mgq, gvSvgvwS mgq) KLb/†Kvb mgq Avcbvi ‡ivRMvi/iæwR fvj nq? ‡Kb? ‡Kvb mgqUv Avcbvi Lvivc hvq? †Kb? hLb GB mgm¨v¸‡jv nq Avcwb wK K‡ib? Avcwb ‡h UvKv †ivRMvi/iæwR K‡ib Zv Li‡Pi e¨vcv‡i †K wm×všÍ †bq? †Kb?

What do you usually do for your livelihood and why? (Probes: dry season, flood period, transition period) In your case when do you earn better and why? When do you feel worse and why? How do you manage when problem arises? Who usually decides how the money you earn will be used? Why?

1. Avcwb †h KvR ¸‡jvi K_v ej‡jb †m¸‡jv QvovI Avcbvi cwiev‡ii Ab¨vb¨ m`m¨iv wK KvR K‡i? (wRÁvmv Kiƒb: †K wK KvR K‡i) †Kb? (wRÁvmv Kiƒb: Pvlvev`/avb Pvl, evwoi mvg‡b dj I kvK-me&wRi evMvb, nuvm gyiMx cvjb, grm Pvl I Ab¨vb¨ KvR m¤ú‡K© †hgb: †bŠKv Pvjv‡bv)

In addition to the above (as mentioned by the participant) what else is done by your household members (ask by whom) and why? (Probes: rice cultivation, vegetables or fruits gardening, poultry or livestock rearing, or fishing or any other activities such as boating)

7. eb¨vi mgq wKfv‡e G KvR¸‡jv K‡ib? (wRÁvmv Kiæb: Pvlvev`/avb Pvl, evwoi mvg‡b dj I kvK-me&wRi evMvb, nuvm gyiMx cvjb BZ¨vw` Kv‡Ri †ÿ‡Î) ïKbvi mgq G KvR¸‡jv K‡ib? hLb cvwb ïKv‡Z _v‡K ZLb wK nq? G iKg K‡e n‡qwQj? ZLb wK K‡iwQ‡jb? (wRÁvmv Kiæb: wKfv‡e cwiKíbv K‡iwQj, wK Pvl K‡iwQj, wKfv‡e exR evQvB K‡iwQj, wKfv‡e Rwg Pvl K‡iwQj, wK iKg djb n‡qwQj, weµq Kiv‡Z †c‡iwQj wKbv? bv cvi‡j ‡Kb cv‡iwb BZ¨vw` m¤ú‡K©)

How do you plan these activities (that we just talked about for example, rice cultivation or gardening, or livestock/poultry rearing etc) given the likelihood during floods. How do you plan these activities in dry season? What happened during transition period? Would you share any relevant experience in this regard? (For agriculture, probes: planning, selection of seeds, cultivation, production and market opportunity)

Avcwb wK Avgv‡K ej‡eb †h Avcwb K…wli gva¨‡g hv hv Drcv`b K‡ib †m¸‡jv w`‡q wK K‡ib/wK Kv‡R jv‡M? (wRÁvmv Kiæb: LvIqv:wK cwigvb Lvq, wK cwigvb Lvq, KZUzKz mvkÖq nq; wewµ Kiv: wewµ Ki‡Z cv‡i wKbv, Avw_©K jvf/ÿwZ) mvavibZ (K„wlKvR ev dm‡ji †ÿ‡Î) Avcbvi wK ai‡bi ÿwZ n‡q _v‡K? K‡e, wKfv‡e Avcbvi G iKg ÿwZ n‡qwQj? (wRÁvmv Kiæb: Avw_©K fv‡e wKfv‡e ÿwZ n‡qwQj, wK cwigvb dmj bó n‡qwQj Ges wKfv‡e, Drcv`b Kg n‡qwQj wKbv, wewµ Ki‡Z Amyweav n‡qwQj wKbv, BZ¨vw` m¤ú‡K©) ZLb Avcwb wK K‡iwQ‡jb (wKfv‡e ÿwZ c~iY K‡iwQ‡jb/ wKfv‡e mvgvj w`‡q‡Qb)?

How do you get benefitted receive through your farming production? (Probes: consumption: how much is consumed, surplus, how the surplus is used market opportunity) What kind of loss do you face usually? Would you share any such experience that you had? How did you mitigate it? (Probes: financial, spoiled, flooded, limited market opportunity, didn’t grow well etc.)

AskMÖnbKvix K…wlKv‡Ri cvkvcvwk Ab¨ †Kvb Kv‡Ri K_v D‡jøL K‡i _vK‡j (†hgb: †bŠKv Pvjv‡bv) GKB fv‡e cÖkœ¸‡jv Kiæb| ‡hgb: (AskMÖnbKvixi D‡jøwLZ) G KvR/¸‡jv K‡i wKfv‡e Avcbvi DcKvi nq? G wel‡q Avcbvi AwfÁZv m¤ú‡K© wKQz e‡jb| (wRÁvmv Kiæb: KvRwU Kivi myweav/Amyweav, Avw_©K jvf/ÿwZ, wKfv‡e ÿwZ c~iY K‡ib BZ¨vw` wel‡q)

If the participant mentions about boating or other activities in addition to farming, ask in the same way, how does it (as mentioned by participant) benefit you? Would you share any example? (Probes: benefits/difficulties, profits/loses, how to mitigate etc)

**Perception on child caring: growth and nutrition:**

**Child Growth:**

Avwg GLb Avcbvi mšÍvb‡`i wel‡q K_v ej‡ev| Zviv †Kgb Av‡Q? Avcbvi me ‡_‡K †QvU mšÍvb m¤ú‡K© wKQz e‡jb| Zvi bvg wK? mvaviYZ †K Zvi (ev”Pvi bvg) †`Lv‡kvbv/ cwiPh©v K‡i? wKfv‡e? †K Zv‡K me ‡_‡K †ekx mvnvh¨ K‡i? wKfv‡e? Zvi (ev”Pvi bvg) †`Lv‡kvbv Kivi Rb¨ Avcwb wK wK K‡ib?

Now let’s talk about your children. How are they doing? Can you talk a bit about the youngest child (ren)? What's his/her name(s)? Who usually takes care of [NAME] and how? Who mostly helps her/him in taking care of the child and how? Can you tell about your own role for his/her caring?

1. GKRb fvj evev nIqvi Rb¨ wK wK ¸b _vKv `iKvi e‡j g‡b K‡ib? (AskMÖnbKvix hZ¸‡jv welq‡K ¸iZ¡c~Y© g‡b K‡ib, me¸‡jv welq‡K we‡ePbvq Avb‡Z n‡e|) Avcbvi wK Ae¯’v?

What kind of qualities/capacities does a father need to have in taking care of his child (Probes: Consider all the important things that the participant consider important). In your case what happens?

1. GKRb fvj gv nIqvi Rb¨ wK wK ¸b _vKv cÖ‡qvRb/`iKvi e‡j g‡b K‡ib? (AskMÖnbKvix hZ¸‡jv welq‡K ¸iZ¡c~Y© g‡b K‡ib, me¸‡jv welq‡K we‡ePbvq Avb‡Z n‡e) Avcbvi (ev”Pvi gv‡qi) wK Ae¯’v?

What kind of qualities/capacities does a mother need to have in taking care of her child (Probes: Consider all the important things that the participant consider important). In your case what happens?

1. Avcbvi wkïi †ÿ‡Î Avcwb wKfv‡e eyS‡Z cv‡ib †h Avcbvi ev”Pv wVKg‡Zv eo wKbv? wK †`‡L/wKfv‡e eyS‡Z cv‡ib †h Avcbvi ev”Pv wVKg‡Zv eo n‡”Q? (wRÁvmv Kiæb: wkï‡K Lvevi,¯^v¯’¨, cwi®‹vi-cwi”QbœZv, ‡Ljva~jv Kiv, Ab¨‡`i mv‡_ †g‡k wKbv A_ev Ab¨ †Kvb welq ‡h¸‡jv AskMÖnbKvix ¸iZ¡c~Y© g‡b K‡ib)

In your case how/when do you feel that your child is growing well or not? What do you do in making sure that your child is growing well? (Probes: food, health, sanitation, hygiene, playfulness, social interaction, or any other contextual issues as considered important by the participant)

1. KLbI wK Ggb n‡qwQj †h Avcbvi ev”Pv wVKg‡Zv eo nw”Qj bv? Avcwb wKfv‡e eyS‡Z ‡c‡iwQ‡jb? (wRÁvmv Kiæb e„w× bv nIqvi wewfbœ kvwiixK jÿb: D”PZv, IRb, Mv‡qi is, Pz‡ji is BZ¨vw`, mvgvwRK I Ab¨vb¨ welq: ‡Ljva~jv bv Kiv, Ab¨‡`i mv‡_ bv †gkv, evievi Amy¯’ nIqv, wLUwL‡U †gRvR BZ¨vw` mn †hme welq AskMÖnbKvix ¸iZ¡c~Y© e‡j g‡b K‡ib) Avgv‡K wK GKUz ej‡eb †h ZLb wK n‡qwQj? wK Kvi‡Y G iKg n‡qwQj e‡j Avcbvi g‡b nq? Avcbvi g‡Z wK Ki‡j G ai‡bi mgm¨v n‡e bv?

Have any of your child ever experienced poor growth? How did you know? (Probes: markers of poor growth i.e. biomedical: height, weight, skin, hair; non-biomedical: playfulness, social interaction, frequent illness, irritability other contextual issues as considered important by the participant). Would you tell the story what happened? What might be the possible reasons for that? How such problem (poor growth) could be prevented?

1. wKQz wKQz wkï‡K j¤^vq Kg/Lv‡Uv g‡b nq| G welqwU‡K Avcwb wKfv‡e †`‡Lb? †Kb GiKg nq? (G wel‡q Zvi fv‡jv ev g›` Abyf‚wZi K_v wRÁvmv Kiæb)

There are some children who are considered to be stunted. What do you think, why it happens?

1. Avevi wKQz wkï‡K ¯^v¯’¨evb ev ‡gvUv‡mvUv g‡b nq| G welqwU‡K Avcwb wKfv‡e †`‡Lb? †Kb? Avevi wKQz ev”Pv LyeB †ivMv-cvZjv nq| G welqwU‡K Avcwb wKfv‡e †`‡Lb? †Kb? (G wel‡q Zvi fv‡jv ev g›` Abyf‚wZi K_v wRÁvmv Kiæb)

Some children are also considered to be fat. How do you see that? Why? On the other hand some children are also considered to be thin. How do you see that? Why? (Probes: good, bad or as you feel)

**Child Nutrition, Health and Interventions:**

1. Avwg GLb ev”Pv‡`i LvIqv-`vIqv I Zv‡`i cywó wel‡q K_v ej‡ev| Avcbvi ev”Pv‡K mvavibZ †K LvIqvq Ges †Kb? mvavibZ Avcbvi ev”Pv‡K wK wK (Lvevi) LvIqv‡bv nq/LIqvb? (wRÁvmv Kiæb: ey‡Ki `ya LvIqv‡bv: KZw`b ïaygvÎ ey‡Ki `ya LvB‡q‡Qb, KZw`b ey‡Ki `ya LvIqv‡eb, evowZ Lvevi LvIqv‡bv: K‡e ïiæ K‡i‡Qb/ Ki‡eb, wK ai‡bi Lvevi †`b, KZevi †`b, Lvev‡i ˆewPÎ¨ m¤ú‡K©)

Now I would like to talk a bit about your child’s feeding and nutrition. Would you tell who usually feed your child and why? Can you tell about the food that are usually offered/feed to your child? (Probes: breastfeeding: initiation, colostrum, duration of exclusive breastfeeding, continuation of breastfeeding, complementary feeding: initiation, type of foods provided, frequency, variation in diets etc., food taboos)

1. Avcbvi ev”Pvi fv‡jv ¯^v¯’¨ I cywói Rb¨ Avcwb mvavibZ wK wK K‡ib ev wK ai‡bi evowZ hZœ †bb? (wRÁvmv Kiæb: cwi¯‹vi-cwi”QbœZv, LvIqv‡bvi Av‡M, b¨vcwKb e`jv‡bvi c‡i, gjg~Î Z¨v‡Mi ci ev”Pv‡K cwi®‹vi Kivi ci nvZ †avqvi Af¨vm m¤ú‡K©, Lvevi wbe©vPb, km¨ Drcv`b, †Ljva~jv, Ab¨‡`i mv‡_ †gj‡gkv Kiv)

What do you usually do/what extra cares do you provide to improve your child’s nutritional status? Probes: cleanliness: hand washing behavior before feeding, during changing of napkins, after defecation, selection of foods, investment for farming production, playing, hanging out, social interaction etc)

1. Avcwb Avcbvi ev”Pv‡K ‡hme Lvevi †L‡Z †`b †m¸‡jv wKfv‡e †hvMvo K‡ib? (wRÁvmv Kiæb: wK‡b Av‡b bvwK Drcv`b K‡i) eb¨vi mgq wK K‡ib? hLb cvwb ïKv‡Z _v‡K H mgqUv‡Z wK K‡ib? (eb¨v/ïKbvi mgq wK ai‡bi Lvevi LvIqv‡Z cv‡ib, wKfv‡e †hvMvo K‡ib, wK ai‡bi Amyweavq ci‡Z nq BZ¨vw` wel‡q wRÁvmv Kiæb) G wel‡q Avcbvi AwfÁZv m¤ú‡K© GKUz e‡jb|

How do you get the foods that you offer to your child? (Probes: purchasing, agricultural production) What happens when there is flood? What do you do when the flood recedes or drought arises? (Usually which food are feed during flood/dry season, how they get those food, problems regarding this issue) Would you share any experience in this regard?

1. †klevi hLb Avcbvi ¯¿xi †c‡U ev”Pv wQj ZLb wZwb wK cwigvb/ wK ai‡bi Lvevi †L‡qwQ‡jb? H mgq Avcwb wK Zvi ‡Kvb evowZ hZœ wb‡qwQ‡jb? wKfv‡e? (wRÁvmv Kiæb: Lvevi, ¯^v¯’¨‡mev, cwiev‡ii hZœ, Kv‡Ri Pvc wel‡q)

Would you tell how much or what type of food was taken by your wife during her last (Name) pregnancy? What kind of extra care was she given during that period? And How? (Probes: Diet, health care, family care, work load).

1. GLb wK Ae¯’v? GLb wKfv‡e Avcwb, Avcbvi cwievi Avcbvi ¯¿xi Avcbvi hZœ †bq? †Kb? (wRÁvmv Kiæb: Lvevi, ¯^v¯’¨‡mev, cwiev‡ii hZœ, Kv‡Ri Pvc wel‡q)

What about now? How do the family care you? Why? (Probes: Diet, health care, family care, work load)

1. Avcbvi g‡Z GKRb gv wKfv‡e wkïi ¯^v¯’¨ I cywói †ÿ‡Î f~wgKv cvjb Ki‡Z cv‡i? Avcbvi ev”Pvi gv wKfv‡e K‡ib?

Would you tell how a mother can contribute in enhancing child’s health and nutrition? In your case what happens?

1. Avcbvi g‡Z GKRb evev wKfv‡e wkïi ¯^v¯’¨ I cywói †ÿ‡Î f~wgKv cvjb Ki‡Z cv‡i? Avcwb wK wK K‡ib?

In your opinion how a father can contribute in enhancing child’s health and nutrition? In your case what happens?

1. Avgiv GLb Avcbvi ev”Pvi ¯^v¯’¨ wel‡q K_v ej‡ev| Avcbvi ev”Pvi kixi ¯^v¯’¨ †Kgb Av‡Q? (wRÁvmv Kiæb: K‡e †m Amy¯’ n‡qwQj, wK n‡qwQj, †Kb) †m Amy¯’ n‡j wK K‡ib? eb¨vi mgq wK K‡ib? ïKbvi mgq ev”Pv Amy¯’ n‡j wK K‡ib? wKfv‡e mgm¨vi mgvavb K‡ib? hLb eb¨vi cvwb ïKv‡Z _v‡K (Pviw`‡K Kv`v _v‡K) †m mgq ev”Pv Amy¯’ n‡j wK K‡ib? Avcbv‡`i †ÿ‡Î KLbI Ggb n‡qwQj? ZLb wK K‡iwQ‡jb?

Let’s talk about your child health. How is his/her health now? (Probes: Did he suffer from any illness, when and why) What did you do for his/her recovery/what do you do when he/she gets sick? What happens when there is flood? How do you solve? And what happens during the transition phase? What about drought? Would share any of your experience in this regard?

1. hLb ev”Pvi gv‡qi ¯^v¯’¨‡mevi cÖ‡qvRb nq ZLb wK K‡ib? (ev”Pvi bvg) hLb †c‡U wQj ZLb wZwb wKfv‡e ¯^v¯’¨‡mev wb‡qwQ‡jb? (ev”Pvi bvg) R‡b¥i mgq wK K‡iwQ‡jb? (†K ¯^v¯’¨‡mev w`‡qwQj, †Kv_vq, †Kb, wPwKrmvi gvb: e¨envi/AvPib,, ch©vß ¯’vb, Av‡M †_‡K Rvbv‡Z nq wK bv, mš‘ó wKbv) eb¨vi mgq wK K‡ib? ïKbvi mgq wKfv‡e wPwKrmv/‡mev ‡bb? gvSvgvwS mg‡q wK nq? G iKg ‡Kvb NUbv wK n‡qwQj? †m m¤ú‡K© Avgv‡K wKQz e‡jb?

What do you do when the mother needs health care? What did you do during her pregnancy and delivery for (NAME)? (Probes: who provided the care, from where, why, quality of care: behavior, appointment issue, satisfaction) How do you manage when there is flood or drought arises or during the transition phase of flood and drought. Would you tell any relevant experience in this regard?

1. hw` Avcbvi wkï ev Lvbv m`m¨ †Kvb miKvix/‡emiKvix cÖwZôvb †_‡K mvnvh¨/mn‡hvMxZv/‡mev †c‡q _v‡Kb, †m m¤ú‡K© wK ej‡eb? (wRÁvmv Kiƒb: ¯^v¯’¨, cwi®‹vi-cwi”QbœZv, K…wl, Avw_©K wel‡q cÖvß mn‡hvMxZv/‡mev m¤ú‡K©) G ‡mevi gva¨‡g wKfv‡e Avcbvi DcKvi n‡q‡Q? wK ai‡bi mn‡hvMxZv/‡mev ‡c‡j Avcbvi AviI †ekx DcKvi n‡Zv e‡j Avcwb g‡b K‡ib?

Would you name the organizations from where you receive any kind of service or help? (Probes: GO/NGOs/BRAC)? Would you tell who does what for you? What kind of services are particularly available for mother and child? (Probes: health, sanitation, hygiene, agriculture, financial) How useful are they? What could have been better?

Avgiv mvÿvrKv‡ii G‡Kev‡i †kl ch©v‡q P‡j G‡mwQ| †kl Kivi Av‡M Avcwb wK AviI wKQz ej‡Z Pvb? mvÿvrKv‡i AskMÖnb K‡i Avcbvi g~j¨evb gZvgZ cÖ`v‡bi Rb¨ ab¨ev` RvbvB|

We already came to the end of the interview, would like to add any other point before we conclude the discussion? Thanks for your valuable inputs and participation.

# **Interview Guides-Focus Group Discussions (FGD)**

## **FGD (Consent Form in Bangla)**

**Introduction**

AvR‡Ki GB `jxq Av‡jvPbvq AskMÖn‡bi Rb¨ Avcbv‡`i mevB‡K ab¨ev` RvbvB| Avgvi bvg................................................. Ges Avgvi mnKvix.................................................| Avgiv Avcbv‡`i Kv‡Q GKwU M‡elbvi Kv‡R G‡mwQ| G M‡elbv Kvh©µgwU †b`vij¨vÛ Gi Groningen University, eª¨vK evsjv‡`k Ges LANSA Gi †hŠ_ D‡Ï¨v‡M, DFID Ges Eric Bleumink Fund (EBF) Gi mn‡hvMxZvq cwiPvwjZ n‡”Q**| G** M‡elbv Kvh©µ‡gi D‡Ïk¨ n‡jv nvIi GjvKvi wkï cywói wewfbœ fv‡jvg›` w`K¸‡jv wbY©q Kiv| GRb¨ Avwg G GjvKvi wkï‡`i ¯^v¯’¨ I cywóMZ Ae¯’v m¤ú‡K© K_v ej‡ev| GB `jxq Av‡jvPbv m¤úbœ Ki‡Z 50-60 wgwbU mgq jvM‡Z cv‡i| Avcbv‡`i m`q AbygwZ mv‡c‡ÿ GB Av‡jvPbv †_‡K cÖvß Z_¨mg~n Avgiv wj‡L wbe Ges ‡iKW© Ki‡ev| G Av‡jvPbvq AskMÖnb Kiv ev bv Kiv m¤ú~Y© Avcbv‡`i B”Qvaxb| Avcbviv ‡h‡Kvb mgq †Kvb Kvib cÖ`k©b QvovB G Av‡jvPbv eÜ Ki‡Z cv‡ib| Z_vwc Avcbv‡`i gZvgZ I `„wófw½ Avgv‡`i Kv‡Q LyeB ¸iZ¡c~Y©| G‡ÿ‡Î mwVK ev fzj DË‡ii ‡Kvb welq †bB| Avcbviv †Kvb cÖ‡kœi DËi w`‡Z ms‡KvP †eva Ki‡j Avgv‡K Rvbv‡Z cv‡ib Ges cÖ‡kœi DËi bv I w`‡Z cv‡ib| Av‡jvPbv PjvKvjxb mgq Avcbv‡`i GKRb GKRb K‡i K_v eivi Rb¨ Aby‡iva KiwQ ‡hb Avcbv‡`i K_vMy‡jv fv‡jvfv‡e †iKW© Kiv hvq|

AvR‡K Avgiv Avcbv‡`i †Kvb †mev ev civgk© ev Avw_©K myweav (UvKv-cqmv) w`‡Z Avwm wb, eis Avcbv‡`i KvQ †_‡K nvI‡ii eZ©gvb Ae¯’v Ges G GjvKvi wkï‡`i ¯^v¯’¨MZ Ae¯’v m¤ú‡K© Rvb‡Z G‡mwQ| Z‡e G Av‡jvPbv †_‡K cÖvß Z_¨ Avcbv‡`i mvwe©K Ae¯’v eyS‡Z mvnvh¨ Ki‡e Ges Avcbv‡`i Ae¯’vi Dbœq‡bi j‡ÿ¨ KZ©„cÿ‡K Rvbv‡bvi †ÿ‡Î mvnvh¨ Ki‡e| G M‡elbvi djvdj ïaygvÎ ˆeÁvwbK Kv‡Ri Rb¨ cÖKvk Kiv n‡e Ges Avcbv‡`i bvg †Kv_vI cÖKvk Kiv n‡e bv| G Av‡jvPbvq AskMÖn‡bi d‡j Avcbvi e¨w³MZ Rxe‡b A_ev eª¨vK ev Ab¨ †Kvb msMVb †_‡K cÖvß †mevi Dci †Kvb Lvivc cÖfve co‡e bv| Avcwb wbwØ©avq GB Av‡jvPbvq AskMÖnb K‡i Avcbvi g~j¨evb gZvZZ Rvbv‡Z cv‡ib | Avcbvi G AskMÖnb Avgv‡`i mK‡ji Kv‡Q cÖkswmZ n‡e| Avcwb wK GB `jxq Av‡jvPbvq AskMÖnb Ki‡Z AvMÖnx ?

**1= n¨uv 2= bv**

AskMÖnbKvix ivRx n‡j Zvi ¯^vÿi wb‡q Av‡jvPbv ïiæ Kiæb|

**AskMÖnbKvixi bvg I ¯^vÿi:**

| **msL¨v** | **bvg** | **¯^vÿi** |
| --- | --- | --- |
|  |  |  |
|  |  |  |
|  |  |  |
|  |  |  |
|  |  |  |
|  |  |  |
|  |  |  |
|  |  |  |

**d¨vwmwj‡UUi/`jxq Av‡jvPbv cwiPvjbvKvixi bvg: ¯^vÿi I ZvwiL:**

**‡bvU †UKvi/ Z_¨ ‡jL‡Ki bvg: ¯^vÿi I ZvwiL:**

## **FGD (Consent Form in English)**

**Introduction**

I would like to thank you all for coming today to this group discussion. My name is ………………………………………… and my assistant is ……………………………………….We came to you to conduct a research project jointly initiated by the BRAC, Bangladesh, Groningen University of the Netherlands and LANSA with financial assistance from DFID and Eric Bleumink Fund (EBF). The main objective of the research project is to understand the issues regarding child nutrition, good or bad, in *haor* areas. For this we would like to have a group discussion on the health and nutrition of the children in *haor* areas. The discussion may take around 50-60 minutes. During the discussion, we would like to take notes and record the discussion if you kindly allow. Your participation is completely voluntary and you may withdraw from the study at any point without giving any reason. However, we value your opinion and views that you will share with us. There is no right or wrong answer. If you feel uncomfortable with any question, you can let us know and can choose not to answer that question. It is requested that only one person talks at a time so that all information shared during this discussion is well recorded.

We haven’t come to you in providing any service or advice or money, rather we came here to know from you about the scenario in *haor*. However, your participation in the research will help us understand the situation and communicate it to relevant stakeholder to improve their efforts. The findings of this research would be published in scientific journal and your name will be kept anonymous. The discussion will not affect the services that you receive from BRAC or others. So you can be comfortable to answer as you feel. We would appreciate if you give us your valuable time for this discussion and freely share your views on the issue. Do you agree?

**1= Yes , 2= No.**

If the participant agrees take his/her signature and continue with the discussion:

**Name and signature of the participant:**

| **S/N** | **Name** | **Signature** |
| --- | --- | --- |
|  |  |  |
|  |  |  |
|  |  |  |
|  |  |  |
|  |  |  |
|  |  |  |
|  |  |  |
|  |  |  |

**FGD facilitator: Signature & Date:**

**Note taker: Signature & Date:**

**FGD Topic guide**

## **FGD Topic Guide**

**FGD No:**

**Dc‡Rjv: MÖvg:**

**cwiwPwZ g~jK cÖkœ (Introductory questions):**

cÖ_‡gB Avgiv cwiwPZ n‡q wbB, Avcbviv G‡Ki ci GK Avcbv‡`i cwiPq w`b| Avcbv‡`i bvg, †ckv wkÿv, †Q‡j‡g‡q‡`i m¤ú‡K© ejyb|

As an introduction, let’s go around so that you can introduce yourselves, and tell us about you**r** name, occupation, education, and children. (Note the background information into the table)

**AskMÖnbKvix‡`i bvg (Participants’ name):**

|  | **eqm**  **Age** | **‡ckv**  **Occupation** | **wkÿv**  **Education** | **wkïi mv‡_ m¤úK©**  **Relationship with child** | **mšÍv‡bi msL¨v**  **Number of Children** | | **2 eQ‡ii Kg eqmx mšÍv‡bi msL¨v**  **Number of children <2 years** | **me †_‡K †QvU mšÍv‡bi eqm**  **Age of youngest child** |
| --- | --- | --- | --- | --- | --- | --- | --- | --- |
|  |  |  |  |  | **RxweZ**  **Alive** | **g„Z**  **lost** |  |  |
| **1** |  |  |  |  |  |  |  |  |
| **2** |  |  |  |  |  |  |  |  |
| **3** |  |  |  |  |  |  |  |  |
| **4** |  |  |  |  |  |  |  |  |
| **5** |  |  |  |  |  |  |  |  |
| **6** |  |  |  |  |  |  |  |  |
| **7** |  |  |  |  |  |  |  |  |
| **8** |  |  |  |  |  |  |  |  |

**Topic 1: Seasonality, agriculture and economic aspects**

‡ivRMvi/iæwR Kivi Rb¨ gvbyl‡K ‡Zv ‡Kvb bv †Kvb KvR Ki‡Z nq| Avcbv‡`i GjvKvq ‡jvKRb mvavibZ wK ai‡bi KvR K‡ib? †Kb? (wRÁvmv Kiæb: cyiæl Ges gwnjviv ‡ivRMv‡ii Rb¨ wK ai‡bi KvR K‡ib)

People usually do some sort of work for earning. In your area what kind of work do people do? Why? (Probes: Earning by women, men).

Avcbv‡`i GjvKvq AvbygvwbK KZRb/KZfvM †jvK K…wlKv‡Ri mv‡_ RwoZ Ges wKfv‡e? (wRÁvmv Kiæb: cyiæl Ges gwnjviv wKfv‡e K…wlKv‡Ri mv‡_ RwoZ, †Kvb ai‡bi K…wl †ekx ‡`Lv hvq: avb Pvl, meRx Pvl, nvm-gyiMx cvjb, Mev`x cï cvjb Ges grm¨ Pvl)

In your area, how many are possibly engaged with agricultural farming and how? (Probes: involvement of men and women, which parts of agriculture are common: rice, vegetables, poultry, livestock and fisheries).

Avgiv Rvwb †h, Avgv‡`i †`‡k wewfbœ FZzz‡Z eb¨v, Liv BZ¨vw` mgm¨v †`Lv †`q Avcbv‡`i GLv‡b wewfbœ FZy/‡gŠmy‡g wK nq? Gi d‡j GB nvIi GjvKvi gvby‡li Rxe‡b wK wK myweav ev Amyweav †`Lv †`q? (wRÁvmv Kiæb: wewfbœ FZz‡Z wK wK ˆewPÎ¨ †`Lv †`q, KLb ïiæ nq, KZw`b _v‡K, Zv‡`i Rxe‡b Gi cÖfve Ges Zvi cwibwZ/djvdj m¤ú‡K©|**)**

We know, there are different type of seasonal periods like flood, drought etc. in our country. What happens in your area? How the variations influence people’s daily life and activities? (Probes: The variations, when it starts, how long it persists, when it recedes, what all aspects it impacts, consequences)

GLb ejyb, Avcbviv eb¨vi mgq wK ai‡bi K…wlKvR K‡i _v‡Kb Ges wKfv‡e? ïKbvi mgq wK ai‡bi K…wlKvR K‡ib Ges wKfv‡e? gvSvgvwS mgqUv‡Z wK K‡ib? G mgq ¸‡jv‡Z †hb (dmj/Drcv`‡bi) ÿwZ bv nq Zvi Rb¨ wK wK K‡ib? (wRÁvmv Kiæb: cwiKíbv, exR evQvB, Pvl, Drcv`b Ges wewµ Kiv m¤ú‡K©)

Would you tell, how do the people plan for their farming activities considering floods? What about the dry season? What do they plan for transition period? Which aspects do they specifically consider in addressing the negative consequences of floods or dry season or transition period? (Probes: planning, selection of seeds, cultivation, production and market opportunity)

eb¨vi mgq G GjvKvi ‡jvKRb wKfv‡e Lvev‡ii e¨e¯’v K‡ib? ïKbvi mgq wK K‡ib? gvSvgvwS mg‡q wKfv‡e K‡ib?

How do the people manage foods during flood? What about the dry season. How do they manage in transition period?

**Topic 2: Child growth and nutrition**

Avgiv Rvwb †h wkï‡`i h‡Zœi cÖ‡qvRb nq| Avcbvi g‡Z, KZ eQi eqm ch©šÍ Zv‡`i hZœ ev cwiPh©vi cÖ‡qvRb nq? wK ai‡bi hZœ ev cwiPh©vi cÖ‡qvRb nq? mvavibZ †K G `vwqZ¡ cvjb K‡i? 2 eQ‡ii Kg eqmx wkï‡`i †ÿ‡Î wK nq? (wRÁvmv Kiæb: ¯^v¯’¨‡mev, Lvevi LvIqv‡bv, cwi®‹vi-cwi”QbœZv, †Ljva~jv Kiv, †eov‡bv BZ¨vw` m¤ú‡K©)

We know that children need some sort of extra care. Till what age do you think children need care? What kind of care do they need? Who usually provides it? What about children below 2 years? (Probes: health care, feeding, sanitation, playing, hanging out etc)

wkïi gv Gi cvkvcwk Lvbvi †Kvb m`m¨/iv wkïi †`Lv‡kvbv K‡ib Ges wKfv‡e? ev”Pvi evev wKfv‡e Zvi †`Lv‡kvbv K‡ib/ hZœ †bq? (wRÁvmv Kiæb: ¯^v¯’¨‡mev, Lvevi LvIqv‡bv, cwi®‹vi-cwi”QbœZv, †Ljva~jv, †eov‡bv BZ¨vw` m¤ú‡K©)

In addition with mother (or principal caregiver), who else of the household members usually participate in child care and how? How does the father get involved in child caring? (Probes: health care, feeding, sanitation, playing, hanging out etc)

eb¨vi mgq G GjvKvi gvby‡liv wK wkï‡`i hZœ ev myiÿvi Rb¨ we‡kl wKQz K‡i? Zviv wKfv‡e GUv K‡i? ïKbvi mgq ev hLb cvwb P‡j hvq (Pviw`‡K Kv`v _v‡K) ZLb ev”Pv‡`i h‡Zœi Rb¨ wK K‡i? †Kb? (wRÁvmv Kiæb: ¯^v¯’¨‡mev, cwi®‹vi-cwi”QbœZv, Lvevi LvIqv‡bv, †Ljva~jv, BZ¨vw` m¤ú‡K©)

What kind of special care do the children need when there is flood? How do the people manage to take care of their children during that period? Do they also need to consider the drought or transition phase and why? (Probes: health care, sanitation, feeding, playing etc.)

GB GjvKvq ev”Pv‡K fvj ivLvi Rb¨/wVKg‡Zv Zvi hZœ †bqvi Rb¨ GKRb gv‡qi wK wK ¸b _vKv `iKvi e‡j g‡b K‡ib? (AskMÖnb Kvixiv †h welq¸‡jv‡K ¸iZ¡c~Y© g‡b K‡ib Zvi me¸‡jvB ¸iZ¡ mnKv‡i we‡ePbv Kiæb, gv‡qi wkÿv I Ávb, my¯’Zv, Avw_©K m¤ú`, wm×všÍ †bqvi ÿgZv, ¯^v¯’¨MZ Ae¯’v BZ¨vw` wel‡q AviI wRÁvmv Kiv †h‡Z cv‡i)|

In this area what kind of qualities/capacities a mother is expected to have in providing good care to the child? (Probes: Consider all the important things that the participant consider important, further probes can be on education and knowledge, healthy behavior, access to financial resources, decision making power, own health status etc.)

GB GjvKvq ev”Pv‡K fvj ivLvi Rb¨/wVKg‡Zv Zvi hZœ †bqvi Rb¨ GKRb evevi wK wK ¸b _vKv `iKvi e‡j g‡b K‡ib? (AskMÖnb Kvixiv †h welq¸‡jv‡K ¸iZ¡c~Y© g‡b K‡ib Zvi me¸‡jvB ¸iZ¡ mnKv‡i we‡ePbv Kiæb, evevi wkÿv I Ávb, my¯’Zv, Avw_©K m¤ú`, wm×všÍ †bqvi ÿgZv, ¯^v¯’¨MZ Ae¯’v, mšÍv‡bi hZœ †bqv ‡ÿ‡Î cÖavb cwiPh©vKvix‡K mn‡hvMxZv Kivi B”Qv BZ¨vw` wel‡q AviI wRÁvmv Kiv †h‡Z cv‡i)|

In this area what kind of qualities/capacities a father is expected to have in providing good care to the child? (Probes: Consider all the important things that the participant consider important, further probes can be on education and knowledge, healthy behavior, access to financial resources, decision making power, own health status, willingness to cooperate the principal caregiver etc.)

GB GjvKvq ev”Pvi eveviv ev”Pvi gv‡qi mv‡_ †Kgb e¨envi K‡ib? †Kb? welqwU‡K G GjvKvi †jvKRb wKfv‡e †`‡L? (wRÁvmv Kiæb: ¯¿xi Mv‡q nvZ †Zvjv/AvNvZ Kiv, MvjvMvwj †`Iqv)? GiKg †Kvb NUbv wK Avgv‡K Rvbv‡eb? hLb G iKg n‡qwQj ZLb Avcbvi ev”Pvi wK †Kvb Amyweav n‡qwQj? (wRÁvmv Kiæb: gv I wkïi gvbwmK Ae¯’v, ev”Pvi LvIqv,)

How do the fathers usually treat the child’s mother in this area and why? How it is justified by the community? (Probes: Beating/hitting wives, abusing etc.) Would you tell or share any relevant story? How does it affect child care?(Probes: emotional, feeding etc.)

Avgiv mevB PvB †h, Avgv‡`i mšÍvb/iv my¯’fv‡e eo ‡nvK| GB GjvKvi gvby‡liv wKfv‡e Rvb‡Z/eyS‡Z cv‡i †h Zvi mšÍvb wVKfv‡e eo n‡”Q bv? (wRÁvmv Kiæb: D”PZv I IRb, LvIqv-`vIqv ev ¯^v¯’¨ msµvšÍ welq, ‡Ljva~jv Kiv, Ab¨‡`i mv‡_ †gjv‡gkv Kiv, ‡gŠmygx cwieZ©b BZ¨vw`) wK wK Kvi‡Y wkï wVKg‡Zv eo nq bv| G iKg †hb bv nq Zvi Rb¨ GjvKvi gvby‡liv wK wK K‡ib?

We all want our children to grow in a healthy way. In this area, how do the people understand whether their child is not growing well? (Probes: anthropometry, feeding or health related aspects, playfulness, social interaction, seasonal change etc.) Which factors do they think responsible for poor growth of a child? What do the people do in preventing poor growth of the child?

G GjvKvi gvbyl wKfv‡e eyS‡Z cv‡i †h Z‡`i ev”Pv wVKfv‡e eo n‡”Q? (c~e©eZ©x DËi¸‡jv Abyhvqx wRÁvmv Kiæb: IRb-&D”PZv, Lvevi LvIqv‡bv, ¯^v‡¯’¨i Ae¯’v, †Ljva~jv Kiv, Ab¨‡`i mv‡_ †gjv‡gkv Kiv BZ¨vw` m¤ú‡K© wRÁvmv Kiæb ) wkï‡`i wVKfv‡e †e‡o DVv wbwðZ Kivi Rb¨ GjvKvi †jvKRb wK wK K‡i?

When do the people feel that their child is growing well? (Continue as it is required according to the previous response, probes: anthropometry, feeding or health related aspects, playfulness, social interaction, seasonal change etc.)? What do the people do in ensuring better growth for the child?

wKQz wkï‡K j¤^vq Lv‡Uv †`Lvq| G welqwU‡K Avcbv‡`i GjvKvi †jvKRb wKfv‡e †`‡L? (Zv‡`i Lvivc ev fv‡jv Abyf~wZ Ges Zvi KviY m¤ú‡K© wRÁvmv Kiæb)

There are some children who look stunted. How do the people think about this? When do the people consider their children stunted? (Probes: good, bad or why)

wKQz wkï‡K LyeB wPKb/cvZjv| G welqwU‡K Avcbv‡`i GjvKvi †jvKRb wKfv‡e †`‡L? Avevi wKQz wkï‡K LyeB ¯^v¯’¨evb/‡gvUv †`Lvq| G welqwU‡K Avcbv‡`i GjvKvi †jvKRb wKfv‡e †`‡L? KLb Zviv ev”Pv‡`i †gvUv ev ïKbv g‡b K‡i (Zv‡`i Lvivc ev fv‡jv Abyf~wZ Ges Zvi KviY m¤ú‡K© wRÁvmv Kiæb)

There are some children who look too thin. How do the people think about this? There are also some children who look too fat. How do the people think about this? When do the people consider their children thin or fat? (Probes: good, bad or why)

Avcbviv Rv‡bb †h, wewfbœ eq‡m wkï‡`i wewfbœ ai‡bi Lvevi w`‡Z nq| Avcbv‡`i GjvKvq gv‡qiv 2 eQi eqm ch©šÍ wkï‡`i mvavibZ wK †L‡Z †`q? (wRÁvmv Kiæb: wewfbœ eq‡m Lvev‡ii aib, cÖwZ †ejvi Lvev‡i wewfbœ& iKg Lv‡`¨i Dcw¯’wZ, Ggb †Kvb Lvevi hv cÖvq me wkï‡K LvIqv‡bv nq, †Kvb we‡kl Lvevi hv mvavibZ wkï‡`i LvIqv‡bv nq bv, †Kvb FZz‡Z wK cvIqv hvq)

You know at different ages we introduce different foods to our children. In your area what do the mothers usually offer to their children to eat, particularly before 2 years of age? (Probes: Type of foods at different ages, diversity in one meal, preference for any specific food, prohibition of any food, seasonal availability etc.)

wKfv‡e Zviv wkïi Lvevi †hvMvo K‡i? (wRÁvmv Kiæb: wK‡b Av‡b bvwK Drcv`b K‡i) eb¨vi mgq wK K‡ib? ïKbvi mgq wK K‡ib? hLb cvwb ïKv‡Z _v‡K H mgqUv‡Z wK K‡ib? (wRÁvmv Kiæb: eb¨v/Livi mgq wK ai‡bi Lvevi LvIqv‡Z cv‡ib, wKfv‡e †hvMvo K‡ib, wK ai‡bi Amyweavq ci‡Z nq BZ¨vw` wel‡q wRÁvmv Kiæb)G wel‡q ‡Kvb NUbvi m¤ú‡K© GKUz e‡jb|

Where do they get the foods for their children? (Probes: purchasing, agricultural production) What happens when there is flood? What happened during dry season? What do you do when the flood recedes or drought arises? (Usually which food are feed during flood/dry season, how they get those food, problems regarding this issue) Would you share any experience in this regard?

**Topic 3: Health care and welfare services**

‡KD Amy¯’ n‡j G GjvKvi ‡jvKRb wK K‡i? Zviv wKfv‡e wPwKrmv Kivb? (wRÁvmv Kiæb: †Kv_vq, Kvi Kv‡Q, KZ `~i, ¯^v¯’¨‡K›`ª Av‡Q wKbv, Wv³v‡ii wfwRU/wdm jv‡M wK bv, Kviv †mev wb‡Z cv‡i, ch©vß ¯’vb Av‡Q wKbv, wPwKrmvi gvb:Wv³vi Av‡Q wKbv, Av‡M †_‡K Rvwb‡q †h‡Z nq wKbv, wmwiqvj w`‡Z nq wKbv, ‡ivMx‡`i mv‡_ †Kgb e¨envi K‡i, ‡ivMx‡K KZÿb mgq †`q, Mªnb‡hvM¨Zv:mv”Q›`¨ †eva K‡i wKbv/‡h †mev cvq Zv‡Z Lykx wKbv)

What do people do when anyone gets sick? How do the people ensure health care? (Probes: where, to whom, how far, affordability, who able to receive the service, availability of space quality of health care: availability of the doctor, appointment issue, hours spent, comfortability).

ev”Pviv Amy¯’ n‡j evev-gv‡qiv wK K‡ib? hLb †Kvb Mf©eZx gv Amy¯’ n‡q c‡ob/cÖm‡ei mgq wK K‡ib? eb¨vi mgq wKfv‡e wPwKrmv Kivb? G mgq wK ai‡bi mgm¨v †`Lv †`q? wKfv‡e Zv †gvKvwejv K‡ib? ïKbvi mgq wKfv‡e wPwKrmv K‡ib? G mgq wK ai‡bi mgm¨vq c‡ob? ZLb wK K‡ib? gvSvgvwS mgq wKfv‡e wPwKrmv K‡ib? wK ai‡bi mgm¨v nq? ZLb wK K‡ib? G iKg †Kvb NUbv wK Avgv‡K ej‡Z cv‡ib? (wRÁvmv Kiæb: Mf©eZx gv, cÖmeKvjxb mgq, nVvr ev”Pv Amy¯’ n‡j, Mvox cvIqv hvq wKbv, `yiZ¡, Wv³vi cvIqv hvq wK bv, ¯^v¯’¨‡K›`ª Av‡Q wK bv, ‡h †mev cvq Zv‡Z Zviv Lykx wKbv BZ¨vw` m¤ú‡K©)

What happens when the child gets sick? What happens when the mother gets sick during pregnancy or delivery? How do people manage when there is flood? What kind of problems usually arise and how do the people address? How do people manage is dry season? What kind of problems usually arise and how do the people address? What happened at the middle between flood and dry season? Can you share any story in this regard? (Probes: Pregnancy, delivery, sudden health problem of a baby, availability of vehicle, distance, availability of doctors or health facility, satisfaction of the patient etc.)

Avcbv‡`i GjvKvi gvbyl Zv‡`i DbœwZi Rb¨ wewfbœ miKvix cÖwZôvb, eª¨vK ev Ab¨vb¨ †emiKvix cÖwZôvb †_‡K Avi wK wK ai‡bi ‡mev †c‡q _v‡Kb? (cÖwZwU ms¯’vi Rb¨ Avjv`v fv‡e wRÁvmv Kiæb) gv I wkïi Rb¨ wK ai‡bi †mev Pvjy Av‡Q? †m¸‡jv ‡Kgb? (wRÁvmv Kiæb: ¯^v¯’¨ †mev, cywó, K…wl, cwi®‹vi-cwi”QbœZv welqK) wK Ki‡j AviI fv‡jv n‡Zv e‡j Avcbviv g‡b K‡ib?

Would you name the organizations that are providing wellbeing services to the people in your area? What kind of services do they provide? (Probes: for each of the organizations)What services are available for mother and children? How are they? (Probes: services on health, nutrition, agriculture, sanitation and hygiene) What would have been better?

Avgiv Av‡jvPbvi G‡Kev‡i †kl ch©v‡q P‡j G‡mwQ| †kl Kivi Av‡M Avcbv‡`i wK AviI wKQz ejvi Av‡Q? `jxq Av‡jvPbvi AskMÖnb K‡i g~j¨evb gZvgZ cÖ`v‡bi Rb¨ Avcbv‡`i AvšÍwiK ab¨ev` RvbvB|

We already came to the end of the discussion, would like to add any other point before we conclude?

Thanks for your valuable inputs and participation.
